# Coordination of care in health systems for users with diabetes and hypertension: a scoping review^*^

**DOI:** 10.1590/1518-8345.7198.4428

**Published:** 2025-01-27

**Authors:** Virgílio Luiz Marques de Macedo, Naira Pereira de Sousa, Ana Cristina dos Santos, Walterlânia Santos, Marina Morato Stival, Tânia Cristina Morais Santa Barbara Rehem

**Affiliations:** 1 Universidade de Brasília, Brasília, DF, Brazil.; 2 Secretaria de Estado de Saúde, Brasília, DF, Brazil.; 3 Escola Superior de Ciências da Saúde, Brasília, DF, Brazil.; 4 Secretaria de Estado de Saúde, Hospital de Base, Brasília, DF, Brazil.

**Keywords:** Hypertension, Diabetes Mellitus, Primary Health Care, Ambulatory Care, Health Systems, Health Evaluation

## Abstract

**Objective:**

to map the available evidence on the characteristics of care coordination between Primary Health Care and Specialized Outpatient Care for users with diabetes and hypertension.

**Method:**

this is a scoping review with 40 articles as the final sample, evaluated by means of Content Analysis, of the thematic-categorical type, with the aid of a technological tool.

**Results:**

care coordination was defined by means of eight categories: information and communication, integration of care, improvement and quality, care management, care sharing, fundamental attribute, health professionals and health service users, with the results of the articles concentrating mainly on four categories, with information and communication standing out, followed by the category of care management and the category of care sharing, in parallel with improvement and quality.

**Conclusion:**

technological tools are a first step in ensuring the coordination of care, proving to be a significant feature, with emphasis on studies on the sharing of information between health services through electronic medical records. However, although this technology has proved to be advantageous for the health system, with good results, it is not the only means of ensuring the coordination of care.

## Highlights

**(1)** Eight categories were identified for the concept of care coordination.

**(2)** The information and communication category stands out.

**(3)** Coordination of care is fundamental for chronic non-communicable diseases.

**(4)** Having a nurse as a care manager can improve care coordination.

## Introduction

Health systems must promote, restore and maintain the health of the population, with a set of services that communicate to guarantee social protection. As a precept of the Unified Health System (SUS), Health Care Networks (HCN) are the future, enabling cooperative work, operationalized in a shared manner and focused on attesting to the quality of care provided^(1-2)^.

For this to happen, the coordination of health care needs to be strengthened, yet this is proving to be a current challenge and one that is causing growing concern. In this sense, the organization of systems and HCNs requires Primary Health Care (PHC) to take on its role as coordinator of care, because when this happens, it proves to be strongly associated with increased access, improved quality of service, user satisfaction and better use of resources^(3-5)^.

The health system, through the coordination of care carried out by PHC, gives users access to Secondary Care (SC), also known as Specialized Outpatient Care (SAC), which is co-responsible for users of the health system, guaranteeing back-up care and consulting on the care provided^(6)^.

It is worth emphasizing that this care needs to be offered in order to guarantee its comprehensive, because only then will it be possible to promote specific assistance when PHC needs a complement. However, in Brazil, the structure of this level of care is insufficient and poorly articulated, causing PHC to work in isolation^(7)^.

It is still known that users with chronic non-communicable diseases (NCDs) need continuous follow-up, because when care is interrupted, there are many losses for the health system and for the user themselves, so effective communication between the various health services is necessary^(8)^.

Systemic arterial hypertension (SAH) and diabetes mellitus (DM) are on the rise in Brazil and worldwide, with an increase in their prevalence in the population and a consequent increase in mortality rates from cardiovascular diseases. This makes it necessary to devise strategies to effectively combat these diseases^(9-11)^.

In this way, coordination of care is relevant since the negative effects of its absence are signs of poor quality health care, and are even more potent in chronic conditions such as SAH and DM^(12)^. Despite this, a study that carried out bibliographic mapping of articles on PHC in Brazil up to 2016^(4)^ found that only 5.5% of the studies were on the attribute of coordination of care in the proportion of articles selected in the sample surveyed by the authors.

In addition, considering the relevance of SAH and DM in PHC, added to the low number of studies on care coordination, as mentioned above, as well as the specificities of care for users with chronic diseases, and considering that it is possible that care coordination is a fundamental strategy for improving the health system, this study aimed to map the available evidence on the characteristics of care coordination between PHC and SAC for users with diabetes and hypertension.

## Method

### Type of study

This is a scoping review conducted in accordance with the methodological guidelines of the Joanna Briggs Institute (JBI) Collaboration for Scoping Reviews, reported in accordance with the Preferred Reporting Items for Systematic Reviews and Meta Analyses extension for Scoping Reviews (PRISMA-ScR) statement^(13)^.

This is a literature review methodology used to map the main concepts, summarize evidence, enable greater breadth of the literature and inform future research. It involves drafting an outline of the review, drawing up inclusion criteria, a search strategy, extracting, presenting and summarizing the results and their implications for research and practice^(13)^.

This methodology was adopted to make it possible to explore the information that is already available on the coordination of care between PHC and SAC in users with SAH and DM, and thus make it possible to analyze the characteristics of this attribute. The review protocol was registered in the Open Science Framework (OSF) under DOI 10.17605/OSF.IO/9CV6N to make the study information public and to encourage transparency and enable the search strategy to be replicated.

### Selection criteria

The guiding question for the review was formulated based on the Population, Concept and Context (PCC) strategy as guided by the JBI, resulting in the following question: “What are the characteristics of the coordination of care for users with diabetes and hypertension between Primary Health Care and Specialized Outpatient Care?”. To construct this question and carry out the search, the PCC strategy was stipulated as: P for users with diabetes and/or hypertension; C for coordination of care; and C for PHC and SAC.

This scoping review considered national and international articles, published in Portuguese, English or Spanish, and did not restrict the date of publication, as the aim of the review was to report on all the existing literature. In addition, it considered articles published in full format, which could be qualitative, quantitative or mixed, and published up to the period of data collection.

Exclusion criteria were defined as review articles because they dealt with the interpretation and synthesis of a non-primary source, letters to the editor, abstracts and papers published in the proceedings of scientific events due to the need for greater robustness of the data presented.

### Data collection

Initially, a test phase was carried out using the PubMed/Medline database to identify articles on the desired topic and analyze the keywords to be used. With the articles referring to the desired topic, it was possible to identify and analyze the words contained to develop the complete search strategy. During the search, no systematic or scoping reviews were found addressing the question of this study.

In order to maximize and guarantee the quality of the research, the searches were carried out with the collaboration of a specialist health sciences librarian, using the Health Sciences Descriptors (DeCS)/Medical Subject Headings (MeSH) and designing Medline medical subject headings related to the research question.

Once the definition had been made and tested, the terms and keywords were adapted to each database, using the search strategy set up for PubMed/Medline as a template: (“Hypertension” [MeSH Terms] OR “Hypertension” [All Fields] OR “High Blood Pressure” [All Fields] OR “High Blood Pressures” [All Fields] OR “Diabetes Mellitus” [MeSH Terms] OR “Diabetes Mellitus” [All Fields] OR “Diabetes” [Title/Abstract]) AND (“Primary Health Care” [MeSH Terms] OR “Primary Health Care” [All Fields] OR “Primary Healthcare” [All Fields] OR “Primary Care” [All Fields] OR “primary- Services” [All Fields] OR ‘Outpatient Services’ [All Fields] OR ‘Outpatient Service’ [All Fields] OR ‘Clinic Visits’ [All Fields] OR ‘Clinic Visit’ [All Fields]) AND (‘Care coordination’ [All fields] OR ‘Coordinated care’ [All fields]).

The search was carried out on April 16, 2023, using institutional access to the CAPES journal portal/CAFe Access (Academic Community), when free and open access was not available. The following information sources were searched: PubMed/MedLine, Embase, Scopus (Elsevier), Web of Science, Cumulative Index to Nursing and Allied Health Literature (CINAHL/EBSCOhost), Latin American and Caribbean Health Sciences Literature (LILACS), Livivo and Google Scholar.

Furthermore, considering that Google Scholar is a source of grey literature, which is not part of official databases and does not follow a rigorous editorial process, the searches tend to show great variability in articles and documents. Therefore, it was decided that only the first 100 search results would be used.

After searching all the databases, the articles found were migrated to the Rayyan^®^ free web version software, enabling automatic and manual analysis of duplicates. With the help of this tool, two researchers were able to blindly and independently carry out the evaluation in two stages: (1) reading the title and abstract and (2) reading the full text. At each stage, any discrepancies were resolved by consensus between the two researchers, as recommended in the literature^(14)^.

Data from the included articles was extracted using a Microsoft Excel^®^ spreadsheet, containing specific information on title, authors, year, country of origin, journal of publication, language, study objective, methodological approach, study population (users with hypertension and diabetes), number of participants, context (PHC and SAC), definition of care coordination and main results.

### Data treatment and analysis

After the data had been extracted, content analysis was used, of the thematic-categorical type, which, according to Minayo^(15)^, this is when we discover the nuclei of meaning of that communication whose frequency or presence means something to the objective of the study. The three recommended stages were also used: pre-analysis, with the organization and preparation of the material; exploration of the material, with the coding of the data through the recording units; and the treatment of the results, interpretation and inferences^(16)^.

For the second stage, exploration of the material, the paid version of the ATLAS.ti Web software was used, which facilitates the creation of categories. The software was used in two stages: first, the content taken from the references was separated into key topics that represented the essence of what was described and that answered the research question. After this, the key topics were aggregated to form a group of categories that were similar, representing the concepts and characteristics of care coordination.

With the categorization carried out using the informed software, it was possible to analyse which categories were most prevalent in each article, cross-referencing the information after processing the results.

### Ethical aspects

Considering that this study did not evaluate human beings, but rather secondary information that is accessible and in the public domain, exclusively through scientific articles, it was not necessary to submit it to the Research Ethics Committee in accordance with National Health Council Resolution No. 466 of December 12, 2012, and its complements.

## Results

**Figure 1:**
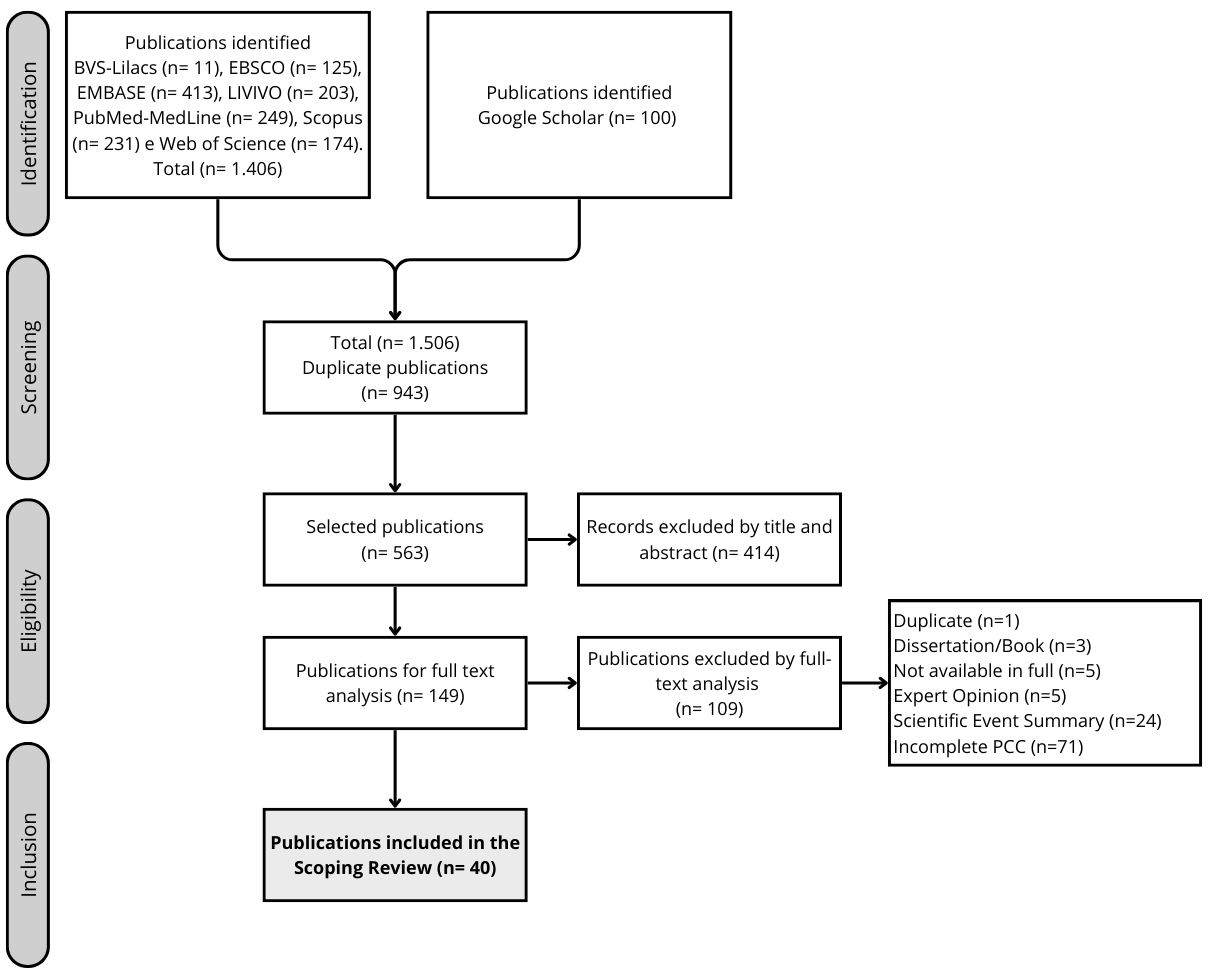
- Flowchart of the process for selecting studies for the scoping review adapted from the Preferred Reporting Items for Systematic Review and Meta-Analyses (PRISMA)^(13)^

The search initially resulted in 1,506 publications, of which 943 were identified as duplicates, leaving 563 publications for reading titles and abstracts. Of these, 149 were eligible for full-text reading, which, after applying the exclusion criteria, resulted in 40 articles included in this review, according to the selection process detailed in Figure 1.

Figure 2 shows the description of the articles that were included as the final sample of this scoping review.

With regard to the year of publication of the selected articles, they date back to the year 2000, with sequential years of publication found from 2009 to 2022. Regarding the country of origin of the articles, they were carried out in the United States (n=25)^(17,19-24,27-29,31-33,35,38,40-43,45-49,53)^, followed by Brazil (n=3)^(36-37,56)^, Australia (n=3)^(30,34,51)^ and the other eight countries with one or two articles: South Africa, Basque Country, Canada, South Korea, India, Italy, Norway and Taiwan.

**Figure 2:** - Box describing the articles selected by author/reference, year, title, methodological approach and population category

**Author / Reference**	**Year**	**Title**	**Methodological approach / Study design**	**Study population**
Schillinger, et al.^(17)^	2000	Effects of primary care coordination on public hospital patients	Quantitative / Randomized	User with hypertension and diabetes
Mills; Harvey^(18)^	2003	Beyond community-based diabetes management and the COAG coordinated care trial	Quantitative / Case Control	User with diabetes
O’Malley; Cunningh^(19)^	2009	Patient experiences with coordination of care: the benefit of continuity and primary care physician as referral source	Quantitative / Cross-sectional study	User with hypertension and diabetes
MacPhailet, et al.^(20)^	2009	Coordination of diabetes care in four delivery models using an electronic health record	Qualitative / Multiple case study	User with diabetes
Liu, et al.^(21)^	2010	Care Fragmentation and Emergency Department Use Among Complex Patients With Diabetes	Quantitative / Observational	User with diabetes
Hummel; Gandara^(22)^	2011	Health information exchange and care coordination of diabetic patients between medicine and dentistry	Qualitative / Case study	User with diabetes
Maeng, et al.^(23)^	2012	Care coordination for the chronically ill: understanding the patient’s perspective	Quantitative / Cross-sectional	User with hypertension and diabetes
Pollack, et al.^(24)^	2013	Patient sharing among physicians and costs of care: a network analytic approach to care coordination using claims data	Quantitative / Cohort	User with diabetes
Baldo, et al.^(25)^	2014	Diabetes outcomes within integrated healthcare management programs	Quantitative / Observational	User with diabetes
Katz, et al.^(26)^	2014	Do primary care physicians coordinate ambulatory care for chronic disease patients in Canada?	Quantitative / Cohort	User with hypertension and diabetes
Liss, et al.^(27)^	2014	Specialty use among patients with treated hypertension in a patient-centered medical home	Quantitative / Time series study	User with hypertension
Segal; Dugoff^(28)^	2014	Building blocks for measuring care coordination with claims data	Qualitative / Observational	User with diabetes
Weeks, et al.^(29)^	2014	Measuring Primary Care Organizational Capacity for Diabetes Care Coordination: The Diabetes Care Coordination Readiness Assessment	Quantitative	User with diabetes
Dawda, et al.^(30)^	2015	Does it matter who organises your health care?	Quantitative	User with diabetes
Haley, et al.^(31)^	2015	Improving Care Coordination Between Nephrology and Primary Care: A Quality Improvement Initiative Using the Renal Physicians Association Toolkit	Quantitative / Longitudinal study	User with hypertension and diabetes
Wang, et al.^(32)^	2015	Association of patient-reported care coordination with patient satisfaction	Quantitative / Cross-sectional study	User with diabetes
Zlateva, et al.^(33)^	2015	Development and validation of the Medical Home Care Coordination Survey for assessing care coordination in the primary care setting from the patient and provider perspectives	Quantitative	User with hypertension and diabetes
Lo, et al.^(34)^	2016	Primary and tertiary health professionals’ views on the health-care of patients with co-morbid diabetes and chronic kidney disease - a qualitative study	Qualitative	User with diabetes
Malkani, et al.^(35)^	2016	Redesigning Diabetes Care: Defining the Role of Endocrinologists Among Alternative Providers	Qualitative	User with diabetes
Venancio, et al.^(36)^	2016	*Atenção integral à hipertensão arterial e diabetes mellitus: implementação da Linha de Cuidado em uma Região de Saúde do estado de São Paulo, Brasil*	Mixed / Case study	User with hypertension and diabetes
Aleluia, et al.^(37)^	2017	*Coordenação do cuidado na atenção primária à saúde: estudo avaliativo em município sede de macrorregião do nordeste brasileiro*	Qualitative / Case study	User with hypertension and diabetes
Fitzgerald, et al.^(38)^	2017	Program Implementation Approaches to Build and Sustain Health Care Coordination for Type 2 Diabetes	Qualitative	User with diabetes
Provost, et al.^(39)^	2017	Implementation of an integrated primary care cardiometabolic risk prevention and management network in Montréal: Does greater coordination of care with primary care physicians have an impact on health outcomes?	Mixed	User with hypertension and diabetes
Talley, et al.^(40)^	2018	Improving Population Health among Uninsured Patients with Diabetes	Quantitative / Observational study	User with diabetes
Van-Eeghen, et al.^(41)^	2018	Chronic care coordination by integrating care through a team-based, population-driven approach: a case study	Qualitative / Case study	User with diabetes
Vimalananda, et al.^(42)^	2018	Patient, Primary Care Provider, and Specialist Perspectives on Specialty Care Coordination in an Integrated Health Care System	Qualitative	User with diabetes
Benzer, et al.^(43)^	2019	Survey of Patient-Centered Coordination of Care for Diabetes with Cardiovascular and Mental Health Comorbidities in the Department of Veterans Affairs	Quantitative / Observational Study	User with hypertension and diabetes
Lee; Bae^(44)^	2019	Implementation of a care coordination system for chronic diseases	Qualitative	User with hypertension and diabetes
McLendo, et al.^(45)^	2019	Enhancing diabetes care through care coordination, telemedicine, and education: Evaluation of a rural pilot program	Quantitative / Cohort	User with diabetes
Mohr et al.^(46)^	2019	Organizational Coordination and Patient Experiences of Specialty Care Integration	Mixed / Cross-sectional study	User with hypertension and diabetes
Williams, et al.^(47)^	2019	Sustainable care coordination: a qualitative study of primary care provider, administrator, and insurer perspectives	Qualitative	User with diabetes
Benzer, et al.^(48)^	2020	A Mixed Methods Study of the Association of Non-Veterans Affairs Care With Veterans’ and Clinicians’ Experiences of Care Coordination	Mixed / Observational study	User with hypertension and diabetes
Harrison, et al.^(49)^	2020	Economic outcomes of insurer-led care management for high-cost Medicaid patients	Quantitative / Cohort	User with hypertension and diabetes
Mateo-Abad, et al.^(50)^	2020	Impact of the CareWell integrated care model for older patients with multimorbidity: a quasi-experimental controlled study in the Basque Country	Mixed / Quasi-experimental study	User with diabetes
Blignault, et al.^(51)^	2021	“You Can’t Work with My People If You Don’t Know How to”: Enhancing Transfer of Care from Hospital to Primary Care for Aboriginal Australians with Chronic Disease	Qualitative	User with diabetes
Chen; Cheng^(52)^	2021	Care Continuity and Care Coordination: A Preliminary Examination of Their Effects on Hospitalization	Quantitative / Cohort	User with diabetes
Cook, et al.^(53)^	2021	Registry-Managed Care Coordination and Education for Patients With Co-occurring Diabetes and Serious Mental Illness	Quantitative / Cohort	User with diabetes
Helmersen, et al.^(54)^	2021	Women’s experience with receiving advice on diet and Self-Monitoring of blood glucose for gestational diabetes mellitus: a qualitative study	Qualitative	User with diabetes
Jindal, et al.^(55)^	2022	Improving care for hypertension and diabetes in India by addition of clinical decision support system and task shifting in the national NCD program: I-TREC model of care	Qualitative / Case study	User with hypertension and diabetes
Rêgo, et al.^(56)^	2022	Coordenação do cuidado na perspectiva das pessoas com hipertensão na atenção primária à saúde	Quantitative / Cross-sectional study	Users with hypertension

The articles were published in 26 different scientific journals, with a higher concentration of articles in the Journal of General Internal Medicine (n=7)^(17,19,24,27,29,43,36)^, followed by the journal BMC Health Services Research (n=5)^(23,33,47,50,55)^. Regarding the language of the articles, they were published in English (n=37)^(17-35,38-55)^ and Portuguese (n=3)^(36-37,56)^. With regard to the methodological approach used in the articles, most of them were defined as quantitative (n=21)^(17-19,21,23-27,29-33,40,43,45,49,52-53,56)^, followed by qualitative (n=14)^(20,22,28,34-35,37-38,41-42,44,47,51,54-55)^ and mixed (n=5)^(36,39,46,48,50)^.

Some definitions of care coordination were identified in the selected articles, so in order to describe them systematized, an analysis of the content was carried out, identifying the most cited characteristics in the text through categorization.

**Figure 3:**
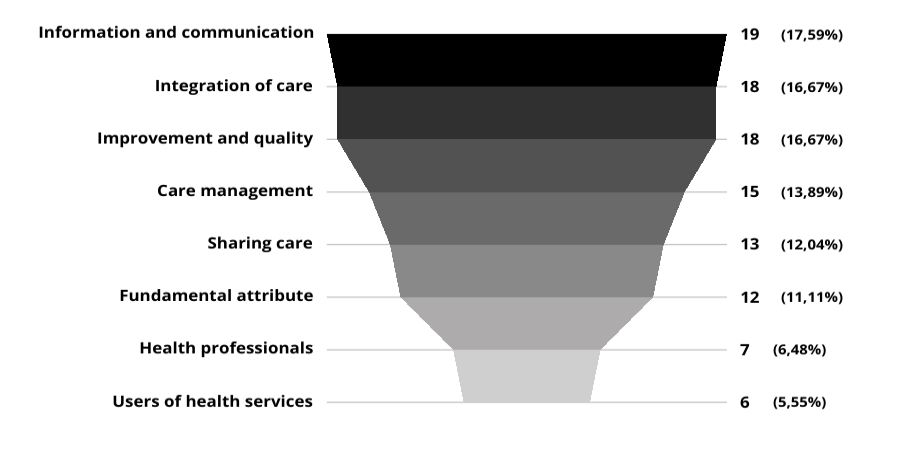
- Funnel graph with definitions of care coordination by category

A total of 108 key topics were listed, divided into 16 categories. However, in order to facilitate understanding, and due to the proximity of the themes, they were grouped into eight categories as shown in the funnel graph in Figure 3, which will be presented below in a summary of each topic, with the characteristics pointed out by the articles, so that the concept of care coordination can be understood in its broad dimension.

### Information and communication

Technological record systems are necessary to enable the recognition and transfer of information between the various service providers, ensuring effective communication in order to facilitate coordinated care, including between the primary care team and other specialists^(22-24,26,28,32-33,38,41,46,48,52-53)^.

### Integration of care

Care coordination is a strategy for providing timely health care, responsible for integrating health services and actions for users in a complementary way within the different levels of care. To this end, it is imperative to structure it, as it represents an essential component in the regionalization of services in health systems^(22,26,28,30,37,47,52,56)^.

### Improvement and quality

Coordinating care means playing a fundamental role in promoting improvements and the appropriate delivery of care to users, promoting an increase in the quality of service provision in an organized, effective and efficient manner. This process leads to improved health outcomes, a reduction in associated costs due to optimized use of services, and significant improvements in clinical results^(20,23,26,32,37-38,43,46-47,53,56)^.

### Care management

In the coordination of care, the planning and organization of assistance can be carried out through the standardization of processes and procedures between the parties. In this sense, managing patients’ needs and monitoring care plans, generating accountability, are essential characteristics for managing the services that users really need, redirecting demands when necessary and anticipating future needs^(19,22,29,33,37-38,41,43,46,52-53,56)^.

### Sharing care

The referral and counter-referral mechanism, in line with best practices in the health area, enables interdependence and the appropriate management of the care offered between the different levels of health services, through a timely and safe transition of care. This process is effective when multiple players, including the user themselves, primary care professionals and other service providers collaborate and share responsibility for care^(20,22-23,29-30,33,37-38,41-42,44,48,52)^.

### Fundamental attribute

Widely recognized as a fundamental attribute, care coordination is of remarkable importance, characterized by being essential to PHC and playing a critical role in promoting access to health services. It ensures continuity of care, minimizing barriers to access and connecting community resources, contributing to the provision of patient-centered care^(19-20,26,30,37-38,52)^.

### Health professionals

A wide variety of health professionals and resources are involved in making care coordination operational. In this sense, in order to carry out the essential activities and care needed by users, it is imperative to establish a solid connection between the parties and to promote efficient work between the teams providing health care^(23,26,37-38,46,53)^.

### Health service users

Although care coordination has been used to serve all users of health services, in the context of chronic conditions, it has emerged as a response strategy that should be encouraged across the board, especially in view of users who receive care from different providers in multiple settings, as this results in an increase in the costs associated with this care and clinical risks^(26,30,32,41,47,56)^.

**Figure 4:**
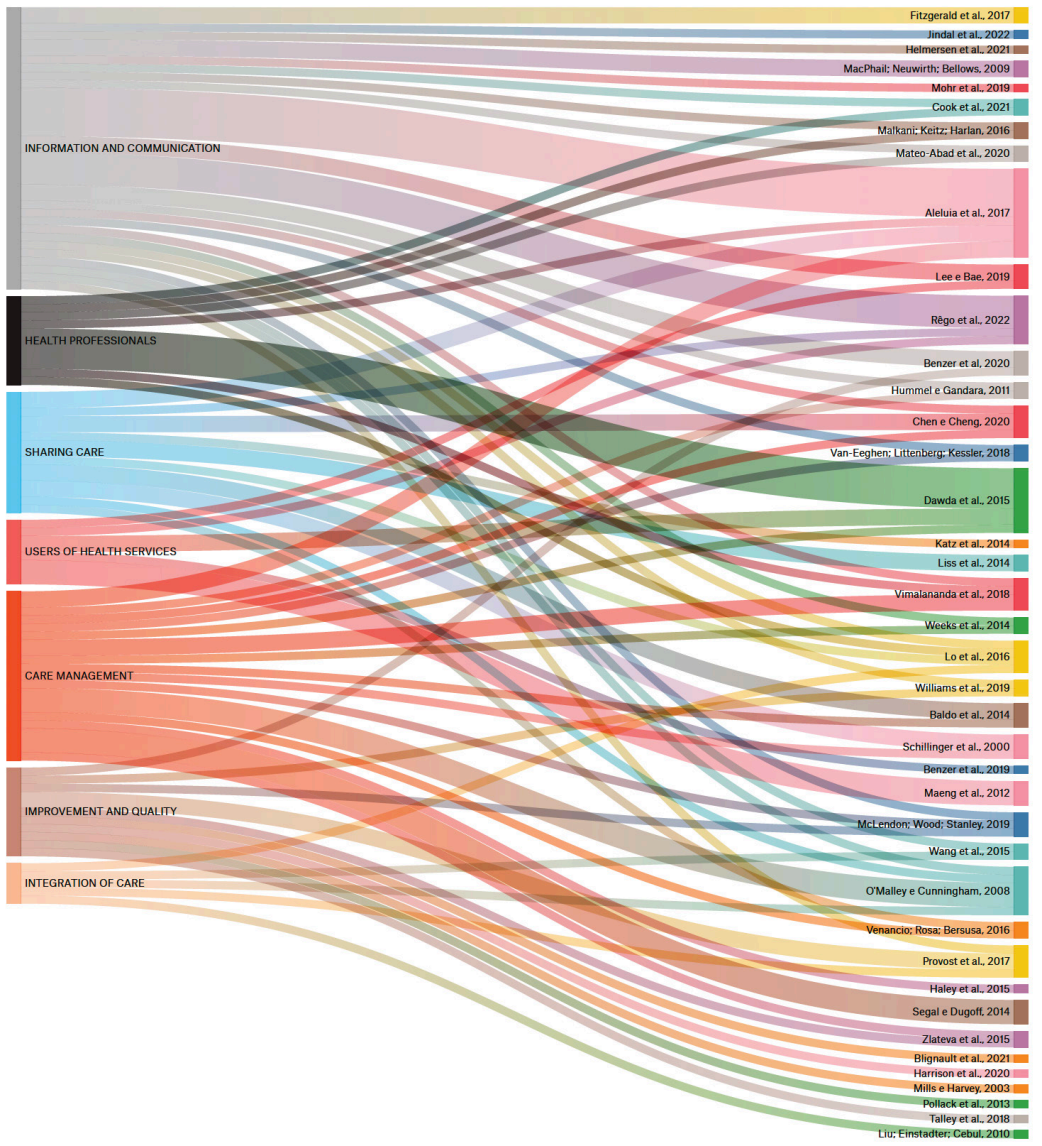
- Sankey diagram of the categories of study results per selected article

With regard to the results presented by the articles, the categories mentioned above were considered, except for the fundamental attribute category, with the aim of relating the main characteristics of the results to the conceptual characteristics pointed out about care coordination. Thus, using the ATLAS.ti Web software, the information identified in the main results of each article was cross-referenced to identify the categories most present in them, using the Sankey Diagram, as shown in Figure 4.

The results of the articles were mainly concentrated in four categories, with the category of information and communication standing out, followed by the category of care management and the category of sharing care in parallel with improvement and quality.

With regard to the information and communication category, this issue has proved to be one of the central challenges of care coordination, since the information provided is not detailed enough for decision-making. However, with the integration of the electronic medical record, information management has been improved and improvements to the electronic medical record, such as signaling feedback, have led to better coordination of care^(22,37-38,46-48,53)^.

In this sense, it was reported that referral through an electronic system simplifies the recording of information and proves to be a common tool for monitoring the user, despite the fact that the user himself, at times, does not identify that the professional is making the notes^(20,45,55-56)^.

On the other hand, the coordination of care requires more than the establishment of electronic resources, and the referral and counter-referral forms have not been very successful, given that they have been little used in counter-referrals^(20,22,29,32,37)^. It is worth noting that an electronic system, depending on the structure required, can be costly for organizations and difficult to implement, mainly due to limited resources^(35,41)^.

In terms of care management, users who are seen by their family doctor and other specialist doctors have better health outcomes than when they are seen by their family doctor alone, reducing hospital use and costs for the health system^(17,25,39,45)^. Also, from the users’ point of view, coordination of care is much better when carried out by a specific primary care professional, and it is also important for this professional to decide which specialist to consult^(19,28)^.

As far as sharing care is concerned, the formal referral and counter-referral mechanisms have proved to be insufficient, mainly due to the lack of counter-referral from the secondary and tertiary levels, including for the treatment of users with diabetes and hypertension. However, the increase in referrals of high morbidity users to specialists highlights the need for more efficient coordination^(27,36-37)^.

It has also been shown that when a doctor is not responsible for the user’s care, they are more likely to make a referral to specialized care due to a lack of information and not knowing the user’s history, which is different from when it is done by primary care professionals^(17,26)^.

With regard to improvement and quality, it was pointed out that the coordination of care should be a priority, since it guarantees the quality of care, generating better health results for users^(18,39,45,48)^. In addition, in order to ensure more appropriate and planned care, the transfer of care must take into account the cultural issues of the users^(51)^.

In this sense, in order to carry out coordinated care with a view to improving the financing of health systems, studies have shown that patients with diabetes and congestive heart failure and those with high risks who received shared care through care coordination had lower total costs, lower hospitalization rates and hospitalization^(24)^.

Another study, although considering that coordination could have reduced total and/or pharmaceutical costs, found no significant evidence^(49)^. Still in relation to costs, it was shown that care coordination, in private health systems, can be charged to users, leading to a lack of user participation due to high costs^(47)^.

Regarding the health professionals category, users and their caregivers perceive that they are good as care coordinators, which is also confirmed by the perception of specialist and primary care doctors^(34,50)^. However, it was found that users with one or more visits to family doctors in the last three months have higher levels of coordination^(30)^.

It was identified, on the other hand, that there are indications that having a nurse as a care manager can improve care coordination, including in the perception of users, who also included an important role for pharmacists and Community Health Workers in this process, mainly through the active search for users during home visits^(30,37,42)^.

In relation to users of health services, those with chronic conditions who consult with the same doctor show an improvement in treatment adherence, with significant results, due to the establishment of a bond and continuity of care^(30,36)^.

In this sense, it was shown that users with diabetes monitored only at the primary level have a higher mortality rate than those shared with the specialized service, with a significant increase in users using insulin^(25)^. However, despite being less likely to be hospitalized, patients with low morbidity are referred in smaller numbers because their needs are met within the scope of primary care^(27,52)^.

Still regarding chronic conditions, as pointed out by the study, when the user presents greater severity, as in cases of type 2 diabetes, fragmentation was more present, suggesting that severity increases coordination challenges^(47)^. Despite this, it has been reported that people with more complex conditions have more consistent care coordination^(30)^.

Studies on users of health services show that those over 60 years of age have higher levels of coordination than those younger, as well as more active users are less likely to report coordination problems because they are better self-managers of their care, and those users who are more complex benefit from greater efforts in terms of care coordination^(23,30)^.

Regarding users with hypertension, those with controlled blood pressure tend to evaluate care coordination positively, while those with decompensated blood pressure evaluate it unsatisfactorily^(56)^. Thus, it is clear that the greater the severity of the patient’s condition, the greater the likelihood that he will need other services and specialties^(44)^.

Regarding the integration of care, greater proximity between teams is necessary in the organizational models of primary care, especially in complex cases of diabetes, in which there is fragmentation of care^(21,39)^. In these situations, continuity of care by the same professionals is beneficial and can even help clarify the roles of professionals in care, which improves care coordination^(19)^.

## Discussion

It is clear that the coordination of care for chronic conditions, specifically for hypertension and diabetes, is a fundamental attribute, generating integration and sharing of care for users, through care management that ensures improvement and quality in care and in the health system, requiring, for its operationalization, excellent means of information, communication and trained professionals.

In this sense, there have been several efforts to investigate and understand the factors, perspectives and challenges that universal health system models have been facing to remain sustainable. However, it is known that hospitals play a leading role and, at the same time, are products of the fragmentation of care. In view of this, it is expected that connections will be produced between health services, including with PHC^(57)^.

It is a fact that all systems face similar challenges in terms of providing effective, efficient and equitable care, even though they have different characteristics and operating methods, as is the case in Brazil and the United States of America (USA). Even so, all have worked to respond to the problems in a way that does not generate economic problems, but rather helps to reduce spending on health services, even with the different ideologies of each system^(58-59)^.

Thus, the coordination of care between PHC and SAC has been discussed even in the USA, which, despite not having a universal health system, tries to identify the experience and perception of users regarding coordination, evaluate the medical results of users with care coordination and assess whether there is a reduction in hospital costs due to coordination^(23-24,48)^.

It is believed that care coordination is responsible for promoting improvements in the quality of care for users, reducing access barriers and integrating actions and services in health systems. Furthermore, there are broad definitions given for this attribute, as pointed out by this study. Thus, it is a fact that the greater the number of people and services involved in care and the more complex the intervention, the greater the level of coordination required to achieve the desired results^(3)^.

In view of the above, it was evident that, in order to ensure full coordination of care, effective information and communication are necessary, and this is achieved through technological record systems with the transfer and interchangeability of user information.

In this sense, the Electronic Health Record (EHR) is understood as a technology that guides the needs of users, uniting the information of an individual or a group and sharing this information between institutions. In addition, the EHR can facilitate health services in monitoring the health situation and financial management through reports. However, the implementation of these systems presents challenges, such as software development, cost-effectiveness, data storage and program performance^(60)^.

A study found that the implementation of the Electronic Patient Record (EPR), used by the PHC in Brazil, favored the organization of the service network with horizontal integration between teams. Additionally, it influenced the co-responsibility of care and the production of autonomy among health professionals^(60)^. The author also reports that there is relevance in using EHR for PHC as it favors care coordination, comprehensiveness of care and longitudinality, which corroborates what was evidenced in the studies found in this review.

However, despite the positive aspects of using EHR, there is still resistance on the part of professionals, which may be related to the lack of training for its use. This was evidenced because, even in places where electronic medical records are already implemented, they are underused^(60)^.

In another study^(61)^, it was reported that some health professionals did not like the idea of implementing electronic medical records, since many did not know how to use them and did not have knowledge about technological equipment. Despite this, with use, they began to have a positive attitude towards the tool, especially with the training provided.

In line with what was reported in the studies of this review, it is understood that EHRs are necessary for the organization and restructuring of a health system, since their absence weakens referral and counter-referral, inhibiting the exchange of information between PHC and other health services. In view of this, it is necessary to integrate medical records in order to access the conduct, examinations and diagnoses performed by professionals so that processes and flows can be managed^(61-62)^.

Regarding care management, the studies found in this review addressed and identified that, in order to coordinate care, it is necessary to plan and organize care with the standardization of processes and procedures.

In view of this, in order to organize health systems, care management is inserted into a new paradigm, since users have complex health needs, requiring greater management capacity. However, this is only possible through relationships between members, with the main focus on the user, with PHC having a complex nature among health services^(63)^.

Therefore, in order to strengthen connections, professionals need to be concerned with health literacy, also known as health literacy, given that users need to process and understand basic information about health, including knowledge about how the health system works and how care is coordinated, including for users with NCDs, as these are highly complex diseases^(64)^.

In this sense, some authors^(65)^ reported that, in the care of chronic conditions, it is necessary to reorganize work processes, with improvements in planning and integration among team members, so that a new way of managing health care can be produced.

In view of this, it is known that, in order to manage the care of users, especially those with chronic conditions, health systems need to strengthen specialized care (outpatient and hospital), which has been one of the biggest problems of the SUS, with insufficient offers to provide what is needed by users^(66)^.

Furthermore, the articles evaluated in this review do not address the role of the SAC in this coordination of care, nor do they address the counter-referral of users with hypertension and diabetes to the PHC. Thus, this is shown to be a gap that may occur because, as demonstrated, the support of the SAC is insufficient, and this barrier has not yet been overcome, which makes further progress impossible.

It is worth noting that the recent regulation published in Brazil, through Ordinance No. 1,604, of October 18, 2023, by the Ministry of Health, which establishes the National Policy for Specialized Health Care, is a guiding milestone for the SUS. In view of this, it is expected that, with the strengthening of the SAC, there will be an improvement in the coordination of care, thus ensuring better support for the PHC.

Regarding the sharing of care, studies in this review showed that, for care coordination to be effective, it is necessary that sharing be done responsibly, generating interdependence and adequate care management. It also highlights that sharing care must occur in a timely and safe manner.

In view of this, a study that addresses the referral of users to cardiologists and endocrinologists concluded that, despite the fact that PHC doctors use referral protocols, adjustments would still be necessary, as these, by themselves, are not capable of solving the problem due to the lack of diagnostic resources^(67)^.

It is worth noting that, with improvements in the implementation of a single medical record system, telecharts and training meetings, care sharing could be adequate, which would avoid unnecessary referrals, reducing queues and costs, and increasing users’ access to SAC^(67)^.

It is important to note that care coordination represents a strategy for integrating levels of care, and is essential for reorienting health services and the needs of users. Therefore, it involves the creation and maintenance of a common structure, which aims to coordinate their interdependence to allow collective work^(68)^.

Furthermore, a study that considered the impact of NCDs on morbidity and mortality and costs for health systems recognized the importance of nursing^(68)^, since nursing professionals are given problem-solving capacity, protagonism and autonomy, as well as coordination capacity, allowing more assertive guidance of users by health services, as pointed out by studies in this review.

In short, it is noted that, with the results presented, the concept and characteristics of care coordination are clearer, which can help to expand the evaluation of this attribute through indicators and improve decision-making, including for the care of users with NCDs. In addition, it is a fact that this attribute needs to be understood, discussed and applied within health systems in all their magnitude.

Thus, this review highlights the importance of care coordination for health systems, and it is clear that coordination has a broad and complex definition. However, some points have proven to be essential for advancing the understanding of this topic, requiring improvements in information and communication, management and sharing of care.

This scoping review has as a limitation the fact that the term “care coordination” is not defined as a DeCS/MeSH descriptor, thus requiring a combination of other descriptors to search for this topic.

Regarding the gaps found, it was observed that there is little information and/or divergent information in the literature regarding the costs of health systems that do or do not have a structure in care coordination. In addition, there is a clear need for more studies that evaluate care coordination from the perspective of SAC, with only the role and challenges of PHC in ensuring that users reach SAC being evident, without addressing care at this level of care and the consequences of this service until users return to PHC.

## Conclusion

This review showed that the studies had as their main results information and communication, care management and care sharing as categories of care coordination. Therefore, it is understood that these are striking characteristics that should be investigated during the evaluation of this attribute.

With technological advances and the speed of information, it is observed that technological tools are a first step in ensuring care coordination, which proved to be a very significant characteristic among the studies in this review, with emphasis on studies on information sharing between health services through electronic medical records. However, although this technology has proven to be very advantageous for the health system, with good results, it is not the only means of ensuring care coordination.

With regard to NCDs, since they are complex diseases that require several professionals to provide adequate care, it is essential that care coordination be effective, since it has proven to be positive and advantageous when implemented. In this sense, it is clear that PHC is the main and most capable service within the health system to carry out care coordination.

However, regarding SAC, more studies are needed to address this health service in relation to care coordination, since weaknesses within the health system have been demonstrated. Furthermore, it is worth noting that PHC alone is not capable of meeting all of the health needs of users, even though it is able to resolve most of the demands.

This scoping review has no conflict of interest and did not receive external funding for its implementation.
