# Supplementary material for: Coordination of care in health systems for users with diabetes and hypertension: a scoping review
Source: Rev Lat Am Enfermagem. 2025 Jan 27;33:e4428. doi: 10.1590/1518-8345.7198.4428 (PMC11774146; doi:10.1590/1518-8345.7198.4428)
Supplement: Translation

# Translation: Coordinación del cuidado en los sistemas de salud para usuarios con diabetes e hipertensión: una revisión de alcance^*^

**DOI:** 10.1590/1518-8345.7198.4427

**Authors:** Virgílio Luiz Marques de Macedo, Naira Pereira de Sousa, Ana Cristina dos Santos, Walterlânia Santos, Marina Morato Stival, Tânia Cristina Morais Santa Barbara Rehem

**Affiliations:** 1 Universidade de Brasília, Brasília, DF, Brasil.; 2 Secretaria de Estado de Saúde, Brasília, DF, Brasil.; 3 Escola Superior de Ciências da Saúde, Brasília, DF, Brasil.; 4 Secretaria de Estado de Saúde, Hospital de Base, Brasília, DF, Brasil.

**Keywords:** Hipertensión, Diabetes Mellitus, Atención Primaria de Salud, Atención Ambulatoria, Sistemas de Salud, Evaluación en Salud

## Abstract

**Objetivo:**

mapear las evidencias disponibles acerca de las características de la coordinación del cuidado entre la Atención Primaria de Salud y la Atención Especializada Ambulatoria para los usuarios con diabetes e hipertensión.

**Método:**

se trata de una revisión de alcance que tuvo 40 artículos como muestra final, evaluados mediante Análisis de Contenido, del tipo temático-categorial, con el auxilio de una herramienta tecnológica.

**Resultados:**

la coordinación del cuidado se definió mediante ocho categorías: información y comunicación, integración del cuidado, mejora y calidad, gestión del cuidado, compartición del cuidado, atributo fundamental, profesionales de la salud y usuarios de los servicios de salud, con una concentración de los resultados de los artículos principalmente en cuatro categorías, destacándose la información y comunicación, seguida por la categoría de gestión del cuidado y la categoría de compartición del cuidado, en paralelo con mejora y calidad.

**Conclusión:**

las herramientas tecnológicas son un primer paso en la garantía de la coordinación del cuidado, demostrando ser una característica significativa, con énfasis en los estudios sobre la compartición de información entre los servicios de salud mediante historiales clínicos electrónicos; sin embargo, a pesar de que esta tecnología se muestra ventajosa para el sistema de salud, con buenos resultados, no es el único medio para garantizar la coordinación del cuidado.

## Destacados

**(1)** Se identificaron ocho categorías para el concepto de la coordinación del cuidado.

**(2)** Destaca la categoría de información y comunicación.

**(3)** La coordinación del cuidado es fundamental para las enfermedades crónicas no transmisibles.

**(4)** Tener a un enfermero como gestor de cuidados puede mejorar la coordinación del cuidado.

## Introducción

Los sistemas de salud deben promover, restaurar y mantener la salud de la población, con un conjunto de servicios que se comunican para garantizar la protección social. Como precepto del Sistema Único de Salud (SUS), las Redes de Atención a la Salud (RAS) son el futuro, permitiendo un trabajo cooperativo, operado de forma compartida y con un enfoque en asegurar la calidad de la atención prestada^(1-2)^.

Para ello, es necesario que la coordinación de la atención en salud esté fortalecida, aunque sigue siendo un desafío actual que genera preocupaciones crecientes. En este sentido, la organización de los sistemas y de las RAS requiere que la Atención Primaria de Salud (APS) asuma su papel como coordinadora del cuidado, pues, cuando esto ocurre, se demuestra que está fuertemente asociada a la ampliación del acceso, mejora en la calidad del servicio prestado, satisfacción de los usuarios y mejor utilización de recursos^(3-5)^.

El sistema de salud, mediante la coordinación del cuidado realizada por la APS, permite a los usuarios acceder a la Atención Secundaria (AS), también denominada Atención Especializada Ambulatoria (AEA), corresponsable de los usuarios del sistema de salud, garantizando el respaldo asistencial y realizando consultorías en relación con los cuidados prestados^(6)^.

Cabe destacar que este cuidado debe ser ofrecido para garantizar su integralidad, pues solo así será posible promover una asistencia específica cuando la APS necesite un complemento. Sin embargo, en Brasil, la estructura de este nivel de atención es insuficiente y poco articulada, lo que lleva a que la APS realice un trabajo aislado^(7)^.

Asimismo, se sabe que los usuarios con enfermedades crónicas no transmisibles (ECNT) necesitan un seguimiento continuo, ya que, cuando el cuidado se interrumpe, se generan varios perjuicios para el sistema de salud y para el propio usuario, siendo necesaria una comunicación eficaz entre los diversos servicios de salud^(8)^.

En lo que respecta a la hipertensión arterial sistémica (HAS) y la diabetes *mellitus* (DM), estas están en aumento en Brasil y en el mundo, con un incremento en su prevalencia en la población y un consecuente aumento en las tasas de mortalidad por enfermedades cardiovasculares. Por lo tanto, es necesario orientar estrategias para el combate efectivo de estas enfermedades^(9-11)^.

De este modo, la coordinación del cuidado se muestra relevante, ya que los efectos negativos de su ausencia son indicativos de una mala calidad en la atención en salud, siendo aún más potentes en las condiciones crónicas, como en la HAS y la DM^(12)^. A pesar de ello, un estudio que realizó un mapeo bibliográfico de artículos sobre la APS en Brasil hasta 2016^(4)^, señaló que solo el 5,5% de los estudios trataban sobre el atributo de la coordinación del cuidado en la proporción de los artículos seleccionados en la muestra levantada por los autores.

Además, considerando la relevancia de la HAS y DM en la APS, sumada al bajo número de estudios sobre la coordinación del cuidado, según lo mencionado anteriormente, así como las especificidades de la asistencia a los usuarios con enfermedades crónicas, y considerando que es posible que la coordinación del cuidado sea una estrategia fundamental para el mejoramiento del sistema de salud, este estudio tuvo como objetivo mapear las evidencias disponibles acerca de las características de la coordinación del cuidado entre la APS y la AEA para los usuarios con diabetes e hipertensión.

## Método

### Tipo de estudio

Se trata de una revisión de alcance conducida de acuerdo con las directrices metodológicas de la Colaboración *Joanna Briggs Institute* (JBI) para Revisiones de Alcance, reportada de acuerdo con la declaración *Preferred Reporting Items for Systematic Reviews and Meta-Analyses extension for Scoping Reviews* (PRISMA-ScR)^(13)^.

Esta es una metodología de revisión de la literatura utilizada para mapear los principales conceptos, resumir evidencias, posibilitar una mayor amplitud de la literatura e informar investigaciones futuras que atraviesa la realización de un esbozo de la revisión, la elaboración de criterios de inclusión, estrategia de búsqueda, extracción, presentación y resumen de los resultados y sus implicaciones para la investigación y la práctica^(13)^.

Se adoptó esta metodología para explorar la información ya disponible sobre la coordinación del cuidado entre la APS y la AEA en usuarios con HAS y DM y, así, posibilitar un análisis de las características de este atributo. El protocolo de revisión fue registrado en el *Open Science Framework* (OSF) bajo el DOI 10.17605/OSF.IO/9CV6N para hacer públicas las informaciones del estudio, estimular la transparencia y permitir que la estrategia de búsqueda de la investigación sea replicada.

### Criterio de selección

La pregunta orientadora de la revisión fue formulada con base en la estrategia Población, Concepto y Contexto (PCC), según lo orientado por la JBI, resultando en la siguiente pregunta: “¿Cuáles son las características de la coordinación del cuidado para usuarios con diabetes e hipertensión entre la Atención Primaria de Salud y la Atención Especializada Ambulatoria?”. Para la construcción de esta pregunta y para la realización de la búsqueda, la estrategia PCC se estipuló como: P para los usuarios con diabetes y/o hipertensión; C para la coordinación del cuidado; y C para la APS y la AEA.

Esta revisión de alcance consideró artículos nacionales e internacionales, publicados en portugués, inglés o español, y no realizó restricciones en cuanto a la fecha de publicación, ya que el objetivo de la revisión fue relatar toda la literatura existente. Además, consideró artículos publicados en su totalidad en formato completo, pudiendo ser cualitativos, cuantitativos o mixtos, y publicados hasta el período de la recolección de datos.

Se definieron como criterios de exclusión los artículos de revisión por abordar la interpretación y síntesis de una fuente no primaria, las cartas al editor, los resúmenes y los trabajos publicados en anales de eventos científicos debido a la necesidad de mayor robustez en los datos presentados.

### Recolección de datos

Inicialmente, se realizó una etapa de prueba mediante la base de datos de *PubMed/Medline* para identificar artículos que abordaran el tema deseado y analizar las palabras clave a ser utilizadas. Con los artículos referentes al tema deseado, fue posible identificar y analizar las palabras contenidas para desarrollar la estrategia de búsqueda completa. Durante la búsqueda, no se encontró ninguna revisión sistemática o de alcance que abordara la cuestión de este estudio.

Para maximizar y garantizar la calidad en la investigación, las búsquedas fueron elaboradas con la colaboración de un bibliotecario especialista en ciencias de la salud, utilizando los Descriptores en Ciencias de la Salud (DeCS)/*Medical Subject Headings* (MeSH) y diseñando encabezados de temas médicos de *Medline* relacionados con la pregunta de investigación.

Con la definición realizada y probada, los términos y palabras clave fueron adaptados a cada base de datos, utilizando como modelo la estrategia de búsqueda montada para *PubMed/Medline*: (“Hypertension” [MeSH Terms] OR “Hypertension” [All Fields] OR “High Blood Pressure” [All Fields] OR “High Blood Pressures” [All Fields] OR “Diabetes Mellitus” [MeSH Terms] OR “Diabetes Mellitus” [All Fields] OR “Diabetes” [Title/Abstract]) AND (“Primary Health Care” [MeSH Terms] OR “Primary Health Care” [All Fields] OR “Primary Healthcare” [All Fields] OR “Primary Care” [All Fields] OR “primary-secondary care interface” [All Fields] OR “collaborative care” [All Fields] OR “Ambulatory Care” [MeSH Terms] OR “Ambulatory Care” [All Fields] OR “Outpatient Care” [All Fields] OR “Outpatient Health Service” [All Fields] OR “Outpatient Health Services” [All Fields] OR “Outpatient Services” [All Fields] OR “Outpatient Service” [All Fields] OR “Clinic Visits” [All Fields] OR “Clinic Visit” [All Fields]) AND (“Care coordination” [All Fields] OR “Coordinated care” [All Fields]).

La búsqueda se realizó el día 16 de abril de 2023, utilizando el acceso institucional del portal de periódicos de la CAPES/Acceso CAFe (Comunidad Académica), cuando no estaba disponible el acceso de forma libre y gratuita. Así, se realizó la búsqueda en las siguientes fuentes de información: *PubMed/MedLine*, *Embas* e, *Scopus* (*Elsevier*), *Web of Science*, *Cumulative Index to Nursing and Allied Health Literature* (CINAHL/EBSCOhost), Literatura Latinoamericana y del Caribe en Ciencias de la Salud (LILACS), *Livivo* y *Google Scholar*.

Además, considerando que *Google Scholar* es una fuente de literatura gris, que no forma parte de bases de datos oficiales y no sigue un proceso editorial riguroso, las búsquedas tienden a encontrar una gran variabilidad de artículos y documentos. Por ello, se definió utilizar solo los primeros 100 resultados de la búsqueda.

Después de la búsqueda en todas las bases de datos, los artículos encontrados fueron migrados al *software Rayyan* ^®^ versión *web* gratuita, posibilitando el análisis automático y manual de duplicados. Con la ayuda de esta herramienta, dos investigadores pudieron, de forma ciega e independiente, realizar la evaluación en dos etapas: (1) lectura del título y resumen y (2) lectura del texto completo. En cada etapa, las divergencias se resolvieron por consenso entre los dos investigadores según lo recomendado en la literatura, sin ser necesario utilizar un tercero^(14)^.

Los datos de los artículos incluidos fueron extraídos usando una hoja de cálculo propia del *software Microsoft Excel* ^®^, que contenía información específica sobre título, autores, año, país de origen, revista de publicación, idioma, objetivo del estudio, enfoque metodológico, población estudiada (usuarios con hipertensión y diabetes), número de participantes, contexto (APS y AEA), definición de coordinación del cuidado y principales resultados.

### Tratamiento y análisis de los datos

Después de que los datos fueron extraídos, se utilizó el Análisis de Contenido, del tipo temático-categorial que, de acuerdo con Minayo^(15)^, se enfoca en descubrir los núcleos de sentido de una comunicación cuya frecuencia o presencia significa algo relevante para el objetivo del estudio. Además, se utilizaron las tres etapas recomendadas: preanálisis, con la organización y preparación del material; exploración del material, con la codificación de los datos mediante las unidades de registro; y el tratamiento de los resultados, interpretación e inferencias^(16)^.

Para la segunda etapa, exploración del material, se utilizó el *software* ATLAS.ti *Web*, versión paga, que facilita la creación de categorías. El uso del *software* se realizó en dos momentos: primero, el contenido extraído de las referencias se separó por temas clave que representaran la esencia de lo descrito y que respondieran a la pregunta de la investigación. Después de esta etapa, los temas clave se agruparon, formando un conjunto de categorías con similitudes, que representaran los conceptos y características de la coordinación del cuidado.

Con la categorización realizada mediante el *software* mencionado, fue posible analizar en cada artículo cuáles categorías fueron más prevalentes, realizando un cruce de las informaciones después del tratamiento de los resultados.

### Aspectos éticos

Considerando que este estudio no realizó evaluaciones con seres humanos, sino con información secundaria de acceso y dominio público, exclusivamente por medio de artículos científicos, no fue necesario someterlo al Comité de Ética en Investigación de acuerdo con la Resolución del Consejo Nacional de Salud nº 466, de 12 de diciembre de 2012, y sus complementarias.

## Resultados

La investigación resultó inicialmente en 1.506 publicaciones, de las cuales 943 fueron identificadas como duplicadas, quedando 563 publicaciones para la lectura de títulos y resúmenes. De estas, 149 fueron elegibles para la lectura del texto completo, lo que, con la aplicación de los criterios de exclusión, resultó en 40 artículos incluidos en esta revisión, según el proceso de selección detallado en la Figura 1.

En la Figura 2 se presenta la descripción de los artículos que fueron incluidos como muestra final de esta revisión de alcance.

En relación con el año de publicación de los artículos seleccionados, estos datan desde el año 2000, con años consecutivos de publicaciones encontradas desde 2009 hasta 2022. En cuanto al país de origen de los artículos, la mayoría fueron realizados en Estados Unidos (n=25)^(17,19-24,27-29,31-33,35,38,40-43,45-49,53)^, seguido de Brasil (n=3)^(36-37,56)^, Australia (n=3)^(30,34,51)^ y otros ocho países con uno o dos artículos, siendo estos: Sudáfrica, País Vasco, Canadá, Corea del Sur, India, Italia, Noruega y Taiwán.

**Figura 1:** - Diagrama de flujo del proceso de selección de estudios para la revisión de alcance adaptado del *Preferred Reporting Items for Systematic Review and Meta-Analyses* (PRISMA)^(13)^

**Figura 2:** - Cuadro de descripción de los artículos seleccionados por autor/referencia, año, título, enfoque metodológico y categoría poblacional

**Autor / Referencia**	**Año**	**Título**	**Enfoque metodológico / Diseño del estudio**	**Población estudiada**
Schillinger, et al.^(17)^	2000	*Effects of primary care coordination on public hospital patients*	Cuantitativo / Aleatorizado	Usuario con hipertensión y diabetes
Mills; Harvey^(18)^	2003	*Beyond community* - *based diabetes management and the COAG coordinated care trial*	Cuantitativo / Caso control	Usuario con diabetes
O’Malley; Cunningh^(19)^	2009	*Patient experiences with coordination of care: the benefit of continuity and primary care physician as referral source*	Cuantitativo / Estudio Transversal	Usuario con hipertensión y diabetes
MacPhailet, et al.^(20)^	2009	*Coordination of diabetes care in four delivery models using an electronic health record*	Cualitativo / Estudio de caso múltiple	Usuario con diabetes
Liu, et al.^(21)^	2010	*Care Fragmentation and Emergency Department Use Among Complex Patients With Diabetes*	Cuantitativo / Observacional	Usuario con diabetes
Hummel; Gandara^(22)^	2011	*Health information exchange and care coordination of diabetic patients between medicine and dentistry*	Cualitativo / Estudio de caso	Usuario con diabetes
Maeng, et al.^(23)^	2012	*Care coordination for the chronically ill: understanding the patient’s perspective*	Cuantitativo / Transversal	Usuario con hipertensión y diabetes
Pollack, et al.^(24)^	2013	*Patient sharing among physicians and costs of care: a network analytic approach to care coordination using claims data*	Cuantitativo / Cohorte	Usuario con diabetes
Baldo, et al.^(25)^	2014	*Diabetes outcomes within integrated healthcare management programs*	Cuantitativo / Observacional	Usuario con diabetes
Katz, et al.^(26)^	2014	*Do primary care physicians coordinate ambulatory care for chronic disease patients in Canada?*	Cuantitativo / Cohorte	Usuario con hipertensión y diabetes
Liss, et al.^(27)^	2014	*Specialty use among patients with treated hypertension in a patient-centered medical home*	Cuantitativo / Estudio de serie temporal	Usuario con hipertensión
Segal; Dugoff^(28)^	2014	*Building blocks for measuring care coordination with claims data*	Cualitativo / Observacional	Usuario con diabetes
Weeks, et al.^(29)^	2014	*Measuring Primary Care Organizational Capacity for Diabetes Care Coordination: The Diabetes Care Coordination Readiness Assessment*	Cuantitativo	Usuario con diabetes
Dawda, et al.^(30)^	2015	*Does it matter who organises your health care?*	Cuantitativo	Usuario con diabetes
Haley, et al.^(31)^	2015	*Improving Care Coordination Between Nephrology and Primary Care: A Quality Improvement Initiative Using the Renal Physicians Association Toolkit*	Cuantitativo / Estudio longitudinal	Usuario con hipertensión y diabetes
Wang, et al.^(32)^	2015	*Association of patient-reported care coordination with patient satisfaction*	Cuantitativo / Estudio Transversal	Usuario con diabetes
Zlateva, et al.^(33)^	2015	*Development and validation of the Medical Home Care Coordination Survey for assessing care coordination in the primary care setting from the patient and provider perspectives*	Cuantitativo	Usuario con hipertensión y diabetes
Lo, et al.^(34)^	2016	*Primary and tertiary health professionals’ views on the health-care of patients with co-morbid diabetes and chronic kidney disease - a qualitative study*	Cualitativo	Usuario con diabetes
Malkani, et al.^(35)^	2016	*Redesigning Diabetes Care: Defining the Role of Endocrinologists Among Alternative Providers*	Cualitativo	Usuario con diabetes
Venancio, et al.^(36)^	2016	*Atenção integral à hipertensão arterial e diabetes mellitus: implementação da Linha de Cuidado em uma Região de Saúde do estado de São Paulo, Brasil*	Mixto / Estudio de caso	Usuario con hipertensión y diabetes
Aleluia, et al.^(37)^	2017	*Coordenação do cuidado na atenção primária à saúde: estudo avaliativo em município sede de macrorregião do nordeste brasileiro*	Cualitativo / Estudio de caso	Usuario con hipertensión y diabetes
Fitzgerald, et al.^(38)^	2017	*Program Implementation Approaches to Build and Sustain Health Care Coordination for Type 2 Diabetes*	Cualitativo	Usuario con diabetes
Provost, et al.^(39)^	2017	*Implementation of an integrated primary care cardiometabolic risk prevention and management network in Montréal: Does greater coordination of care with primary care physicians have an impact on health outcomes?*	Mixto	Usuario con hipertensión y diabetes
Talley, et al.^(40)^	2018	*Improving Population Health among Uninsured Patients with Diabetes*	Cuantitativo / Estudio observacional	Usuario con diabetes
Van-Eeghen, et al.^(41)^	2018	*Chronic care coordination by integrating care through a team-based, population-driven approach: a case study*	Cualitativo / Estudio de Caso	Usuario con diabetes
Vimalananda, et al.^(42)^	2018	*Patient, Primary Care Provider, and Specialist Perspectives on Specialty Care Coordination in an Integrated Health Care System*	Cualitativo	Usuario con diabetes
Benzer, et al.^(43)^	2019	*Survey of Patient-Centered Coordination of Care for Diabetes with Cardiovascular and Mental Health Comorbidities in the Department of Veterans Affairs*	Cuantitativo / Estudio Observacional	Usuario con hipertensión y diabetes
Lee; Bae^(44)^	2019	*Implementation of a care coordination system for chronic diseases*	Cualitativo	Usuario con hipertensión y diabetes
McLendo, et al.^(45)^	2019	*Enhancing diabetes care through care coordination, telemedicine, and education: Evaluation of a rural pilot program*	Cuantitativo / Cohorte	Usuario con diabetes
Mohr et al.^(46)^	2019	*Organizational Coordination and Patient Experiences of Specialty Care Integration*	Mixto / Estudio transversal	Usuario con hipertensión y diabetes
Williams, et al.^(47)^	2019	*Sustainable care coordination: a qualitative study of primary care provider, administrator, and insurer perspectives*	Cualitativo	Usuario con diabetes
Benzer, et al.^(48)^	2020	*A Mixed Methods Study of the Association of Non-Veterans Affairs Care With Veterans’ and Clinicians’ Experiences of Care Coordination*	Mixto / Estudio Observacional	Usuario con hipertensión y diabetes
Harrison, et al.^(49)^	2020	*Economic outcomes of insurer-led care management for high-cost Medicaid patients*	Cuantitativo / Cohorte	Usuario con hipertensión y diabetes
Mateo-Abad, et al.^(50)^	2020	*Impact of the CareWell integrated care model for older patients with multimorbidity: a quasi-experimental controlled study in the Basque Country*	Mixto / Estudio cuasiexperimental	Usuario con diabetes
Blignault, et al.^(51)^	2021	*“You Can’t Work with My People If You Don’t Know How to”: Enhancing Transfer of Care from Hospital to Primary Care for Aboriginal Australians with Chronic Disease*	Cualitativo	Usuario con diabetes
Chen; Cheng^(52)^	2021	*Care Continuity and Care Coordination: A Preliminary Examination of Their Effects on Hospitalization*	Cuantitativo / Cohorte	Usuario con diabetes
Cook, et al.^(53)^	2021	*Registry-Managed Care Coordination and Education for Patients With Co-occurring Diabetes and Serious Mental Illness*	Cuantitativo / Cohorte	Usuario con diabetes
Helmersen, et al.^(54)^	2021	*Women’s experience with receiving advice on diet and Self-Monitoring of blood glucose for gestational diabetes mellitus: a qualitative study*	Cualitativo	Usuario con diabetes
Jindal, et al.^(55)^	2022	*Improving care for hypertension and diabetes in India by addition of clinical decision support system and task shifting in the national NCD program: I-TREC model of care*	Cualitativo / Estudio de caso	Usuario con hipertensión y diabetes
Rêgo, et al.^(56)^	2022	*Coordenação do cuidado na perspectiva das pessoas com hipertensão na atenção primária à saúde*	Cuantitativo / Estudio Transversal	Usuario con hipertensión

Los artículos fueron publicados en 26 revistas científicas diferentes, con una mayor concentración en la revista *Journal of General Internal Medicine* (n=7)^(17,19,24,27,29,43,36)^, seguida por la revista BMC *Health Services Research* (n=5)^(23,33,47,50,55)^. En cuanto al idioma de los artículos, la mayoría fueron publicados en inglés (n=37)^(17-35,38-55)^ y en portugués (n=3)^(36-37,56)^. En lo que respecta al enfoque metodológico utilizado en los artículos, la mayoría se definieron como cuantitativos (n=21)^(17-19,21,23-27,29-33,40,43,45,49,52-53,56)^, seguidos por cualitativos (n=14)^(20,22,28,34-35,37-38,41-42,44,47,51,54-55)^ y mixtos (n=5)^(36,39,46,48,50)^.

Algunas definiciones de coordinación del cuidado fueron identificadas en los artículos seleccionados. Por lo tanto, para describirlas de manera sistematizada, se realizó un análisis del contenido abordado, identificando en el texto las características más citadas mediante la categorización.

Se enumeraron 108 temas clave, divididos en 16 categorías. Sin embargo, para facilitar la comprensión y dada la cercanía de las temáticas, se agruparon en ocho categorías, como muestra el gráfico de embudo en la Figura 3. A continuación, se presentará una síntesis de cada tema, con las características señaladas por los artículos, para que el concepto de coordinación del cuidado pueda ser entendido en su amplia dimensión.

**Figura 3:** - Gráfico de embudo con las definiciones de la coordinación del cuidado por categorías

### Información y comunicación

Los sistemas de registros tecnológicos son necesarios para viabilizar el reconocimiento y la transferencia de información entre los diversos proveedores de servicios, asegurando una comunicación eficaz, con el objetivo de facilitar el cuidado coordinado, incluso entre el equipo de atención primaria y los demás especialistas^(22-24,26,28,32-33,38,41,46,48,52-53)^.

### Integración del cuidado

La coordinación del cuidado es una estrategia de prestación de servicios de salud oportuna, responsable de integrar los servicios y las acciones de salud de los usuarios de manera complementaria dentro de los diferentes niveles de atención. Para ello, es imperativa su estructuración, ya que representa un componente esencial en la regionalización de los servicios en los sistemas de salud^(22,26,28,30,37,47,52,56)^.

### Mejora y calidad

Coordinar el cuidado significa desempeñar un papel fundamental en la promoción de mejoras y en la entrega adecuada de cuidados a los usuarios, promoviendo un aumento en la calidad de la prestación de servicios de manera organizada, eficaz y eficiente. Este proceso propicia la mejora de los resultados en el área de la salud, la reducción de los costos asociados debido a la optimización de la utilización de servicios y mejoras significativas en los resultados clínicos^(20,23,26,32,37-38,43,46-47,53,56)^.

### Gestión del cuidado

En la coordinación del cuidado, la planificación y organización de la asistencia pueden llevarse a cabo mediante la estandarización de procesos y procedimientos entre las partes. En este sentido, gestionar las necesidades de los pacientes y monitorear los planes de cuidado, generando responsabilidad, son características esenciales para que se gestionen los servicios que los usuarios realmente necesitan, reorientando las demandas cuando sea necesario y anticipando las necesidades futuras^(19,22,29,33,37-38,41,43,46,52-53,56)^.

### Compartición del cuidado

El mecanismo de referencia y contrarreferencia, en conformidad con las mejores prácticas en el área de la salud, viabiliza la interdependencia y la adecuada conducción del cuidado ofrecido entre las distintas instancias de servicios de salud, mediante una transición de cuidado oportuna y segura. Este proceso se efectúa cuando múltiples intervinientes, incluyendo el propio usuario, los profesionales de atención primaria y los demás proveedores de servicios, colaboran y comparten la responsabilidad de los cuidados^(20,22-23,29-30,33,37-38,41-42,44,48,52)^.

### Atributo fundamental

Ampliamente reconocida como un atributo fundamental, la coordinación del cuidado tiene una notable importancia, caracterizándose por ser esencial para la APS y por desempeñar un papel crítico en la promoción del acceso a los servicios de salud. Asegura la continuidad del cuidado, minimizando barreras de acceso y estableciendo la conexión entre los recursos comunitarios, contribuyendo a la provisión de cuidados centrados en el paciente^(19-20,26,30,37-38,52)^.

### Profesionales de la salud

Existe la participación de una extensa variedad de profesionales de la salud y recursos para operacionalizar la coordinación del cuidado. En este sentido, para que la ejecución de las actividades esenciales y los cuidados necesarios para los usuarios sean viables, es imperativo el establecimiento de una sólida conexión entre las partes y la promoción de un trabajo eficiente entre los equipos prestadores de cuidados en salud^(23,26,37-38,46,53)^.

### Usuarios de los servicios de salud

Aunque la coordinación del cuidado se ha empleado para atender a todos los usuarios de los servicios de salud, en el contexto de las condiciones crónicas, surge como una estrategia de respuesta que debe ser estimulada en toda su amplitud, especialmente en virtud de los usuarios que reciben cuidados de diversos proveedores en múltiples entornos, ya que esto resulta en un incremento en los costos asociados a esos cuidados y en riesgos clínicos^(26,30,32,41,47,56)^.

En lo que respecta a los resultados presentados por los artículos, se consideraron las categorías mencionadas anteriormente, excepto la categoría de atributo fundamental, con el objetivo de relacionar las principales características de los resultados con las características conceptuales señaladas sobre la coordinación del cuidado. Así, mediante el *software* ATLAS.ti *Web*, se realizó el cruce de la información identificada en los principales resultados de cada artículo para identificar las categorías más presentes, utilizando el Diagrama de Sankey, según la Figura 4.

**Figura 4:** - Diagrama de Sankey de las categorías de los resultados de los estudios por artículo seleccionado

Los resultados de los artículos se concentraron principalmente en cuatro categorías, destacándose la categoría de *información y comunicación*, seguida por la categoría de *gestión del cuidado* y la categoría de *compartición del cuidado*, en paralelo con *mejora y calidad*.

En lo que respecta a la categoría de *información y comunicación*, esta temática se ha mostrado como uno de los desafíos centrales de la coordinación de cuidados, ya que la información proporcionada no presenta detalles suficientes para la toma de decisiones. Sin embargo, con la integración del historial clínico electrónico, la gestión de la información ha mejorado y, además, mejoras en el historial clínico electrónico, como la señalización de *feedback*, generaron una mejora en la coordinación del cuidado^(^ ^22^ ^,^ ^37^ ^-^ ^38^ ^,^ ^46^ ^-^ ^48^ ^,^ ^53^ ^)^.

En este sentido, se informó que el envío por intermedio de un sistema electrónico simplifica el registro de la información y se muestra como una herramienta útil para el seguimiento del usuario, aunque el propio usuario, en algunos momentos, no identifique que el profesional está realizando las anotaciones^(^ ^20^ ^,^ ^45^ ^,^ ^55^ ^-^ ^56^ ^)^.

Por otro lado, la *coordinación del cuidado* requiere más que el establecimiento de recursos electrónicos; además, las fichas de referencia y contrarreferencia no han tenido mucho éxito, ya que se han utilizado poco en la contrarreferencia^(^ ^20^ ^,^ ^22^ ^,^ ^29^ ^,^ ^32^ ^,^ ^37^ ^)^. Cabe destacar que un sistema electrónico, dependiendo de la estructura necesaria, puede tener costos elevados para las organizaciones, siendo de difícil implementación principalmente debido a los recursos limitados^(^ ^35^ ^,^ ^41^ ^)^.

En cuanto a la *gestión del cuidado*, los usuarios que tienen atención con el médico de familia y otros médicos especialistas presentan mejores resultados de salud que cuando son atendidos solo por el médico de familia, reduciendo la utilización hospitalaria y los costos para el sistema de salud^(^ ^17^ ^,^ ^25^ ^,^ ^39^ ^,^ ^45^ ^)^. Además, desde el punto de vista de los usuarios, la coordinación del cuidado es mucho mejor cuando la realiza un profesional de atención primaria específico, siendo también importante la decisión de este profesional sobre qué especialista consultar^(^ ^19^ ^,^ ^28^ ^)^.

En lo que respecta a la *compartición del cuidado*, los mecanismos formales de referencia y contrarreferencia han demostrado ser insuficientes, principalmente debido a la ausencia de contrarreferencia de los niveles secundarios y terciarios, incluso para el tratamiento de usuarios con diabetes e hipertensión. Sin embargo, el aumento en la derivación de usuarios con alta morbilidad a especialistas destaca la necesidad de una coordinación más eficiente^(^ ^27^ ^,^ ^36^ ^-^ ^37^ ^)^.

Se evidenció, además, que cuando un médico no es el responsable del cuidado del usuario, es más probable que realice una derivación a la atención especializada por falta de información y por no conocer el historial del usuario, lo cual difiere de cuando la derivación es realizada por los profesionales de atención primaria^(^ ^17^ ^,^ ^26^ ^)^.

En cuanto a la *mejora y calidad*, se señaló que la coordinación del cuidado debe ser una prioridad, ya que garantiza la calidad de la atención, generando mejores resultados de salud para los usuarios^(^ ^18^ ^,^ ^39^ ^,^ ^45^ ^,^ ^48^ ^)^. Además, para garantizar una atención más adecuada y planificada, la transferencia del cuidado debe tener en cuenta las cuestiones culturales de los usuarios^(^ ^51^ ^)^.

En este sentido, para realizar un cuidado coordinado desde la perspectiva de la mejora del financiamiento de los sistemas de salud, estudios evidenciaron que pacientes con diabetes e insuficiencia cardíaca congestiva, y aquellos con altos riesgos que recibieron cuidados compartidos mediante la coordinación del cuidado, presentaron menores costos totales, menor tasa de internación y hospitalización^(^ ^24^ ^)^.

Otro estudio, aunque consideró que la coordinación podría haber reducido los costos totales y/o farmacéuticos, no encontró evidencias significativas^(^ ^49^ ^)^. Además, en cuanto a los costos, se evidenció que la coordinación del cuidado en sistemas de salud privados puede ser cobrada a los usuarios, lo que conduce a la falta de participación de estos debido a los altos costos^(^ ^47^ ^)^.

En lo que concierne a la categoría de *profesionales de la salud*, los usuarios y sus cuidadores perciben que estos son buenos coordinadores del cuidado, lo que también fue ratificado por la percepción de los médicos especialistas y de atención primaria^(^ ^34^ ^,^ ^50^ ^)^. No obstante, se señaló que los usuarios con una o más visitas a los médicos de familia en los últimos tres meses tienen niveles de coordinación más elevados^(^ ^30^ ^)^.

Se identificó, por otro lado, que existen indicios de que tener a un enfermero como gestor de cuidados puede mejorar la coordinación del cuidado, incluso en la percepción de los usuarios, quienes también destacaron el papel importante de los farmacéuticos y de los Agentes Comunitarios de Salud en este proceso, principalmente mediante la búsqueda activa de los usuarios en las visitas domiciliarias^(^ ^30^ ^,^ ^37^ ^,^ ^42^ ^)^.

En relación con los *usuarios de los servicios de salud*, aquellos con condiciones crónicas que se consultan con el mismo médico muestran una mejora en la adherencia al tratamiento, con resultados significativos, debido al establecimiento de un vínculo y a la continuidad del cuidado^(^ ^30^ ^,^ ^36^ ^)^.

En este sentido, se evidenció que los usuarios con diabetes que son atendidos solo en el nivel primario tienen tasas de mortalidad más altas que aquellos compartidos con el servicio especializado, con un aumento significativo en los usuarios que utilizan insulina^(^ ^25^ ^)^. Sin embargo, a pesar de tener menor probabilidad de ser hospitalizados, los pacientes con baja morbilidad son derivados en menor cantidad debido a que sus necesidades se satisfacen en el ámbito de la atención primaria^(^ ^27^ ^,^ ^52^ ^)^.

Asimismo, en lo que respecta a las condiciones crónicas, según lo señalado por un estudio, cuando el usuario presenta mayor gravedad, como en los casos de diabetes tipo 2, la fragmentación estaba más presente, lo que sugiere que la gravedad aumenta los desafíos de la coordinación^(^ ^47^ ^)^. A pesar de ello, se informó que las personas con condiciones más complejas tienen una coordinación del cuidado más consistente^(^ ^30^ ^)^.

Estudios sobre los usuarios de los servicios de salud muestran que aquellos mayores de 60 años tienen niveles de coordinación más elevados que los de menor edad, así como los usuarios más activos son menos propensos a reportar problemas de coordinación, ya que son mejores autogestores de sus cuidados, y aquellos usuarios que son más complejos se benefician de mayores esfuerzos en cuanto a la coordinación del cuidado^(^ ^23^ ^,^ ^30^ ^)^.

En relación con los usuarios con hipertensión, aquellos con presión arterial controlada tienden a evaluar positivamente la coordinación del cuidado, mientras que los descompensados la evalúan de manera insatisfactoria^(^ ^56^ ^)^. Así, se percibe que, cuanto mayor es la gravedad de la condición del paciente, mayor es la probabilidad de que necesite otro servicio y especialidades^(^ ^44^ ^)^.

Respecto a la *integración del cuidado*, es necesaria una mayor proximidad entre los equipos en los modelos organizativos de la atención primaria, especialmente en los casos complejos de diabetes, donde existe fragmentación del cuidado^(^ ^21^ ^,^ ^39^ ^)^. En estas situaciones, la continuidad de la atención por los mismos profesionales es beneficiosa y puede favorecer, incluso, la clarificación de los roles de los profesionales en el cuidado, lo que mejora la coordinación del cuidado^(^ ^19^ ^)^.

## Discusión

Es evidente que la coordinación del cuidado para las condiciones crónicas, específicamente para la HAS y la DM, se caracteriza por ser un atributo fundamental, generando integración y compartición de los cuidados de los usuarios mediante una gestión del cuidado que garantiza la mejora y calidad en la asistencia y en el sistema de salud, necesitando para su operacionalización excelentes medios de información, comunicación y profesionales capacitados.

En este sentido, se han realizado numerosos esfuerzos para investigar y comprender los factores, perspectivas y desafíos que los modelos de sistemas de salud universales están enfrentando para mantenerse sostenibles. Sin embargo, se sabe que los hospitales tienen un papel protagonista y, al mismo tiempo, son productos de la fragmentación del cuidado. Frente a esto, se espera que se generen conexiones entre los servicios de salud, incluyendo la APS^(57)^.

Es un hecho que todos los sistemas enfrentan desafíos similares en cuanto a la atención efectiva, eficiente y equitativa, incluso presentando diferentes características y formas de operación, como en el caso de Brasil y los Estados Unidos de América (EE.UU.). Aun así, todos han trabajado para responder a los problemas de una manera que no genere problemas económicos, sino que ayude a reducir los gastos en los servicios de salud, a pesar de las diferentes ideologías de cada sistema^(58-59)^.

Así, la coordinación del cuidado entre la APS y la AEA se está discutiendo incluso en los EE.UU., que, aunque no tienen un sistema de salud universal, intentan identificar la experiencia y percepción de los usuarios frente a la coordinación, evaluar los resultados médicos de los usuarios con la coordinación del cuidado y evaluar si hay disminución de los costos hospitalarios debido a la coordinación^(23-24,48)^.

Se cree que la coordinación del cuidado es responsable de promover mejoras en la calidad de la asistencia a los usuarios, reducir las barreras de acceso e integrar las acciones y servicios en los sistemas de salud. Además, las definiciones dadas para este atributo son amplias, como se señaló en este estudio. Así, es un hecho que, cuanto mayor sea la cantidad de personas y servicios involucrados en el cuidado y más compleja sea la intervención, mayor será el nivel de coordinación necesario para alcanzar los resultados deseados^(3)^.

Ante lo expuesto, se evidenció que, para garantizar una coordinación del cuidado en su totalidad, es necesaria una información y comunicación efectivas, lo que se realiza mediante sistemas de registros tecnológicos con la transferencia e intercambio de la información de los usuarios.

En este sentido, se entiende el Registro Electrónico de Salud (RES) como una tecnología que orienta las necesidades de los usuarios, uniendo la información de un individuo o de un grupo y compartiendo esa información entre las instituciones. Además, el RES puede facilitar los servicios de salud en el monitoreo de la situación de salud y la gestión financiera mediante informes. Sin embargo, la implementación de estos sistemas presenta desafíos, como el desarrollo de *software*, la relación costo-efectividad, el almacenamiento de datos y el desempeño de los programas^(60)^.

Un estudio realizado descubrió que la implementación del Prontuario Electrónico del Paciente (PEP), utilizado por la APS en Brasil, favoreció la organización de la red de servicios con integración horizontal entre los equipos. Además, influyó en la corresponsabilización del cuidado y en la producción de la autonomía de los profesionales de salud^(60)^. El autor también señala que el uso del PEP en la APS es relevante, ya que favorece la coordinación del cuidado, la integralidad de la asistencia y la longitudinalidad, lo que corrobora lo evidenciado en los estudios encontrados en esta revisión.

No obstante, a pesar de los aspectos positivos del uso del RES, todavía existe resistencia por parte de los profesionales, lo cual podría estar relacionado con la falta de capacitación para su uso. Esto se evidenció porque, incluso en los lugares donde el prontuario electrónico ya está implementado, se encuentra subutilizado^(60)^.

En otro estudio^(61)^, se informó que algunos profesionales de la salud no estaban a favor de implementar el prontuario electrónico, ya que muchos no sabían cómo utilizarlo y no tenían conocimientos sobre equipos tecnológicos. A pesar de ello, con el uso, comenzaron a tener una actitud positiva hacia la herramienta, especialmente después de las capacitaciones realizadas.

En consonancia con lo que se informó en los estudios de esta revisión, se comprende que los RES son necesarios para la organización y reestructuración de un sistema de salud, ya que su ausencia debilita la referencia y contrarreferencia, inhibiendo el intercambio de información entre la APS y otros servicios de salud. Ante esto, es necesario integrar los registros del prontuario para acceder a las conductas, exámenes y diagnósticos realizados por los profesionales, de modo que se pueda gestionar los procesos y flujos^(61-62)^.

En cuanto a la gestión del cuidado, los estudios encontrados en esta revisión abordaron e identificaron que, para llevar a cabo la coordinación del cuidado, es necesario el planeamiento y la organización de la asistencia con la estandarización de procesos y procedimientos.

Por lo tanto, para organizar los sistemas de salud, la gestión del cuidado se inserta en un nuevo paradigma, ya que los usuarios han presentado necesidades de salud complejas, requiriendo una mayor capacidad de gestión. Sin embargo, esto solo es posible mediante las relaciones entre los integrantes, con un enfoque principal en el usuario, considerando que la APS tiene un carácter complejo entre los servicios de salud^(63)^.

De esta forma, para fortalecer las conexiones, los profesionales deben preocuparse por la literacidad para la salud, también conocida como alfabetización en salud, ya que los usuarios necesitan procesar y comprender la información básica sobre salud, incluyendo el conocimiento sobre el funcionamiento del sistema de salud y la coordinación del cuidado, especialmente para los usuarios con enfermedades crónicas no transmisibles (ECNT), debido a la elevada complejidad de estas enfermedades^(64)^.

En este sentido, algunos autores^(65)^ señalaron que, en la atención a las condiciones crónicas, es necesaria la reorganización de los procesos de trabajo, con mejoras en la planificación e integración entre los miembros del equipo, para que se produzca un nuevo modelo de gestión del cuidado en salud.

Ante esto, se sabe que, para llevar a cabo la gestión del cuidado de los usuarios, especialmente aquellos con condiciones crónicas, los sistemas de salud necesitan una atención especializada (ambulatoria y hospitalaria) fortalecida, lo que ha sido uno de los mayores problemas del SUS, con ofertas insuficientes para satisfacer las necesidades de los usuarios^(66)^.

Además, los artículos evaluados en esta revisión no abordan el papel de la AEA en esta coordinación del cuidado, ni se menciona la contrarreferencia de los usuarios con hipertensión y diabetes hacia la APS, lo que representa una laguna, posiblemente porque, como se ha demostrado, el apoyo de la AEA es insuficiente y aún no se ha superado esta barrera, lo que impide otros avances.

Cabe destacar que la reciente normativa publicada en Brasil, mediante la *Portaria* nº 1.604, de 18 de octubre de 2023, del Ministerio de Salud, que instituye la Política Nacional de Atención Especializada en Salud, es un marco orientador para el SUS. Ante esto, se espera que, con el fortalecimiento de la AEA, se produzca una mejora en la coordinación del cuidado, garantizando así un mejor apoyo a la APS.

En cuanto a la compartición del cuidado, los estudios de esta revisión indicaron que, para que la coordinación del cuidado sea efectiva, es necesario que la compartición se realice con responsabilidad, generando interdependencia y una adecuada conducción del cuidado. También se destaca que la compartición del cuidado debe realizarse de manera oportuna y segura.

En vista de esto, un estudio que aborda la derivación de usuarios al cardiólogo y al endocrinólogo concluyó que, aunque los médicos de la APS utilizan los protocolos de derivación, aún serían necesarias adecuaciones, ya que estos, por sí solos, no son capaces de resolver el problema debido a la falta de recursos diagnósticos^(67)^.

Se subraya que, con mejoras en la implementación de un sistema de prontuario único, *telecharts* y reuniones de capacitación, la compartición del cuidado podría ser adecuada, lo que evitaría derivaciones innecesarias, reduciría filas y costos, y aumentaría el acceso de los usuarios a la AEA^(67)^.

Se destaca que la coordinación del cuidado representa una estrategia para la integración de los niveles de atención, siendo imperativa para reorientar los servicios de salud y las necesidades de los usuarios. Ante esto, implica la creación y el mantenimiento de una estructura común, que tiene el propósito de coordinar su interdependencia para permitir un trabajo colectivo^(68)^.

Además, un estudio que consideró el impacto de las ECNT en la morbimortalidad y los costos para los sistemas de salud, reconoció la importancia de la enfermería^(68)^, ya que se otorga al profesional de enfermería la capacidad resolutiva, protagonismo y autonomía, así como la capacidad de coordinación, permitiendo una orientación más precisa de los usuarios a través de los servicios de salud, como lo indicaron estudios de esta revisión.

En resumen, se observa que, con los resultados presentados, el concepto y las características de la coordinación del cuidado se vuelven más claros, lo que puede ayudar a ampliar la evaluación de este atributo por medio de indicadores y mejorar la toma de decisiones, incluso para la atención de usuarios con ECNT, además de que es un hecho que este atributo debe ser comprendido, discutido y aplicado en los sistemas de salud en toda su magnitud.

Por lo tanto, esta revisión evidencia la importancia de la coordinación del cuidado para los sistemas de salud, así como deja claro que la coordinación posee una definición amplia y compleja, sin embargo, algunos puntos resultaron esenciales para el avance en la comprensión de este tema, siendo necesario el perfeccionamiento de la información y la comunicación, la gestión y el compartir del cuidado.

Esta revisión de alcance presenta como limitación el hecho de que el término “coordinación del cuidado” no está definido como un descriptor DeSC/MeSH, siendo necesario para la búsqueda de este tema una combinación de otros descriptores.

En cuanto a las lagunas encontradas, se observó que hay poca información y/o información divergente en la literatura con relación a los costos de los sistemas de salud que presentan o no estructuración en la coordinación del cuidado. Además, es notable la necesidad de más estudios que evalúen la coordinación del cuidado desde la perspectiva de la AEA, siendo evidente solo el papel y los desafíos de la APS para que los usuarios lleguen a la AEA, sin abordar el cuidado en este nivel de atención ni los desdoblamientos de este servicio hasta el retorno de los usuarios a la APS.

## Conclusión

Esta revisión mostró que los estudios tuvieron como principales resultados la información y comunicación, la gestión del cuidado y el compartir del cuidado como categorías de la coordinación del cuidado, ante esto, se entiende que son características destacadas que deben ser investigadas durante la evaluación de este atributo.

Con los avances tecnológicos y la rapidez de la información, se observa que las herramientas tecnológicas son un primer paso en la garantía de la coordinación del cuidado, lo que resultó ser una característica muy significativa entre los estudios de esta revisión, con énfasis en los estudios sobre el compartir de información entre los servicios de salud mediante historiales electrónicos. Sin embargo, aunque esta tecnología ha demostrado ser muy ventajosa para el sistema de salud, con buenos resultados, no es el único medio para garantizar la coordinación del cuidado.

En cuanto a las ECNT, al tratarse de enfermedades complejas que requieren de diversos profesionales para brindar un cuidado adecuado, es fundamental que la coordinación del cuidado sea efectiva, ya que ha demostrado ser positiva y ventajosa cuando se lleva a cabo. En este sentido, es evidente que la APS es el servicio principal y más capacitado dentro del sistema de salud para realizar la coordinación del cuidado.

No obstante, en relación con la AEA, se necesitan más estudios que aborden este servicio de salud en cuanto a la coordinación del cuidado, ya que ha demostrado fragilidades dentro del sistema de salud. Además, se destaca que la APS, por sí sola, no es capaz de resolver todas las necesidades de salud de los usuarios, aunque resuelve en gran parte las demandas.

Esta revisión de alcance no presenta conflicto de intereses y no recibió financiamiento externo para su realización.

# Translation: Coordenação do cuidado nos sistemas de saúde a usuários com diabetes e hipertensão: uma revisão de escopo^*^

**DOI:** 10.1590/1518-8345.7198.4429

**Authors:** Virgílio Luiz Marques de Macedo, Naira Pereira de Sousa, Ana Cristina dos Santos, Walterlânia Santos, Marina Morato Stival, Tânia Cristina Morais Santa Barbara Rehem

**Affiliations:** 1 Universidade de Brasília, Brasília, DF, Brasil.; 2 Secretaria de Estado de Saúde, Brasília, DF, Brasil.; 3 Escola Superior de Ciências da Saúde, Brasília, DF, Brasil.; 4 Secretaria de Estado de Saúde, Hospital de Base, Brasília, DF, Brasil.

**Keywords:** Hipertensão, Diabetes *Mellitus*, Atenção Primária à Saúde, Assistência Ambulatorial, Sistemas de Saúde, Avaliação em Saúde

## Abstract

**Objetivo:**

mapear as evidências disponíveis acerca das características da coordenação do cuidado entre a Atenção Primária à Saúde e a Atenção Especializada Ambulatorial aos usuários com diabetes e hipertensão.

**Método:**

trata-se de uma revisão de escopo que teve 40 artigos como amostra final avaliados por meio de Análise de Conteúdo, do tipo temático **-** categorial, com auxílio de ferramenta tecnológica.

**Resultados:**

a coordenação do cuidado foi definida por meio de oito categorias: informação e comunicação, integração do cuidado, melhoria e qualidade, gestão do cuidado, compartilhamento do cuidado, atributo fundamental, profissionais da saúde e usuários dos serviços de saúde, com concentração dos resultados dos artigos principalmente em quatro categorias, destacando-se a informação e comunicação, seguida da categoria de gestão do cuidado e da categoria de compartilhamento do cuidado, em paralelo com melhoria e qualidade.

**Conclusão:**

ferramentas tecnológicas são um primeiro passo na garantia da coordenação do cuidado, mostrando-se uma característica significativa, com ênfase nos estudos sobre o compartilhamento de informações entre os serviços de saúde por meio de prontuários eletrônicos. No entanto, apesar dessa tecnologia se mostrar vantajosa para o sistema de saúde, com bons resultados, não é o único meio de se garantir a coordenação do cuidado.

## Destaques

**(1)** Foram identificadas oito categorias para o conceito da coordenação do cuidado.

**(2)** Destaque para categoria de informação e comunicação.

**(3)** A coordenação do cuidado é fundamental para as doenças crônicas não transmissíveis.

**(4)** Ter um enfermeiro como gestor de cuidados pode melhorar a coordenação do cuidado.

## Introdução

Os sistemas de saúde devem promover, restaurar e manter a saúde da população, como um conjunto de serviços que se comunicam, para garantir a proteção social. Como preceito do Sistema Único de Saúde (SUS), as Redes de Atenção à Saúde (RAS) são o futuro, possibilitando um trabalho cooperativo, operacionalizado, de forma compartilhada e com foco em atestar a qualidade da assistência prestada^(1-2)^.

Para isso, é necessário que a coordenação da atenção à saúde esteja fortalecida, no entanto, tal fato tem se mostrado um desafio atual e que gera preocupações crescentes. Nesse sentido, a organização dos sistemas e das RAS demanda que a Atenção Primária à Saúde (APS) assuma seu papel como coordenadora do cuidado, pois, quando isso ocorre, ela demonstra estar fortemente associada à ampliação do acesso, melhora na qualidade do serviço prestado, satisfação aos usuários e melhor utilização de recursos^(3-5)^.

O sistema de saúde, por meio da coordenação do cuidado realizada pela APS, possibilita aos usuários acesso à Atenção Secundária (AS), também denominada Atenção Especializada Ambulatorial (AEA), corresponsável pelos usuários do sistema de saúde, garantindo a retaguarda assistencial e realizando consultorias frente aos cuidados prestados^(6)^.

Vale ressaltar que o cuidado precisa ser ofertado para garantir a sua integralidade, pois somente assim será possível promover assistência específica quando a APS necessitar de um complemento. No entanto, no Brasil, a estrutura desse nível de atenção é insuficiente e pouco articulada, fazendo com que a APS realize um trabalho isolado^(7)^.

Ainda se sabe que os usuários com doença crônica não transmissível (DCNT) precisam de um acompanhamento contínuo, pois, quando o cuidado é interrompido, vários são os prejuízos para o sistema de saúde e para o próprio usuário, sendo necessária uma comunicação eficaz entre os diversos serviços de saúde^(8)^.

No que se refere à hipertensão arterial sistêmica (HAS) e a diabetes *mellitus* (DM), elas estão em ascensão no Brasil e no mundo, com aumento da sua prevalência na população e consequente aumento das taxas de mortalidade por doenças cardiovasculares. Isto posto, faz-se necessário o direcionamento de estratégias para o combate efetivo dessas doenças^(9-11)^.

Desse modo, a coordenação do cuidado se mostra relevante, já que os efeitos negativos de sua ausência são sinais de má qualidade da atenção à saúde, sendo ainda mais potentes nas condições crônicas, como na HAS e na DM^(12)^. Apesar disso, um estudo que realizou mapeamento bibliográfico de artigos sobre a APS no Brasil até 2016^(4)^ apontou que somente 5,5% dos estudos foram sobre o atributo da coordenação do cuidado, na proporção dos artigos selecionados na amostra levantada pelos autores.

Além disso, considerando a relevância da HAS e DM na APS somada ao número baixo de estudos sobre a coordenação do cuidado, conforme supracitado, bem como as especificidades da assistência aos usuários com doenças crônicas, e também considerando que é possível que a coordenação do cuidado seja uma estratégia fundamental para o aprimoramento do sistema de saúde, este estudo teve como objetivo mapear as evidências disponíveis acerca das características da coordenação do cuidado entre a APS e a AEA aos usuários com diabetes e hipertensão.

## Método

### Tipo de estudo

Trata-se de uma revisão de escopo conduzida de acordo com as diretrizes metodológicas da Colaboração *Joanna Briggs Institute* (JBI) para revisão de escopo, relatada de acordo com a declaração *Preferred Reporting Items for Systematic Reviews and Meta Analyses extension for Scoping Reviews* (PRISMA-ScR)^(13)^.

Esta é uma metodologia de revisão de literatura utilizada para mapear os principais conceitos, resumir evidências, possibilitar maior amplitude da literatura e informar pesquisas futuras que perpassam a realização de um esboço da revisão, a elaboração de critérios de inclusão, estratégia de busca, extração, apresentação e resumo dos resultados e suas implicações para a pesquisa e para a prática^(13)^.

Esta metodologia foi adotada para possibilitar explorar as informações que já estão disponíveis acerca da coordenação do cuidado entre a APS e a AEA em usuários com HAS e DM e, assim, possibilitar uma análise das características desse atributo. O protocolo de revisão foi registrado no *Open Science Framework* (OSF) sob o DOI 10.17605/OSF.IO/9CV6N para deixar público as informações do estudo e estimular a transparência e possibilitar que a estratégia de busca da pesquisa seja replicada.

### Critérios de seleção

A questão norteadora da revisão foi formulada com base na estratégia População, Conceito e Contexto (PCC) conforme orientado pela JBI, resultando na seguinte questão: “Quais as características da coordenação do cuidado a usuários com diabetes e hipertensão entre a Atenção Primária à Saúde e a Atenção Especializada Ambulatorial?”. Para a construção desta questão e para a realização da busca, a estratégia PCC foi estipulada como: P para os usuários com diabetes e/ou hipertensão; C para coordenação do cuidado; e C para APS e AEA.

Esta revisão de escopo considerou artigos nacionais e internacionais, publicados em português, inglês ou espanhol, e não realizou restrição de data de publicação, pois o objetivo da revisão foi relatar toda a literatura existente. Além disso, considerou-se artigos publicados na íntegra em formato completo, podendo ser qualitativos, quantitativos ou mistos, e publicados até o período da coleta de dados.

Foram definidos como critérios de exclusão os artigos de revisão, por abordarem a interpretação e síntese de uma fonte não primária, as cartas ao editor, os resumos e os trabalhos publicados em anais de eventos científicos devido à necessidade de maior robustez dos dados apresentados.

### Coleta de dados

Inicialmente, foi realizada uma etapa-teste por meio da base de dados da *PubMed*/*Medline* para identificar artigos que abordassem o tema desejado e analisar as palavras-chave a serem utilizadas. Com os artigos referentes ao tema desejado, foi possível identificar e analisar as palavras contidas para desenvolver a estratégia de busca completa. Durante a busca, nenhuma revisão sistemática ou de escopo que abordasse a questão deste estudo foi encontrada.

Para maximizar e garantir a qualidade na pesquisa, as buscas foram elaboradas com a colaboração de bibliotecário especialista em ciências da saúde, utilizando os Descritores em Ciências da Saúde (DeCS)/*Medical Subject Headings* (MeSH) e projetando cabeçalhos de assuntos médicos da *Medline* relacionados à pergunta da pesquisa.

Com a definição realizada e testada, os termos e palavras-chave foram adaptados a cada base de dados, utilizando como modelo a estratégia de busca montada para a *PubMed*/*Medline*: (“Hypertension” [MeSH Terms] OR “Hypertension” [All Fields] OR “High Blood Pressure” [All Fields] OR “High Blood Pressures” [All Fields] OR “Diabetes Mellitus” [MeSH Terms] OR “Diabetes Mellitus” [All Fields] OR “Diabetes” [Title/Abstract]) AND (“Primary Health Care” [MeSH Terms] OR “Primary Health Care” [All Fields] OR “Primary Healthcare” [All Fields] OR “Primary Care” [All Fields] OR “primary-secondary care interface” [All Fields] OR “collaborative care” [All Fields] OR “Ambulatory Care” [MeSH Terms] OR “Ambulatory Care” [All Fields] OR “Outpatient Care” [All Fields] OR “Outpatient Health Service” [All Fields] OR “Outpatient Health Services” [All Fields] OR “Outpatient Services” [All Fields] OR “Outpatient Service” [All Fields] OR “Clinic Visits” [All Fields] OR “Clinic Visit” [All Fields]) AND (“Care coordination” [All Fields] OR “Coordinated care” [All Fields]).

A busca foi realizada no dia 16 de abril de 2023, utilizando-se do acesso institucional do portal de periódicos da CAPES/Acesso CAFe (Comunidade Acadêmica), quando não disponível o acesso de forma livre e gratuita. Assim, foi realizada busca nas seguintes fontes de informação: *PubMed*/*MedLine*, Embase, *Scopus* (*Elsevier*), *Web of Science*, *Cumulative Index to Nursing and Allied Health Literature* (CINAHL/*EBSCOhost*), Literatura Latino-Americana e do Caribe em Ciências da Saúde (LILACS), Livivo e *Google Scholar*.

Ademais, considerando que o *Google Scholar* é uma fonte de literatura cinzenta, que não faz parte de dados oficiais e não segue um processo editorial rigoroso, as buscas realizadas tendem a apresentar grande variabilidade de artigos e documentos. Por isso, foi definido que somente os 100 primeiros resultados da busca seriam utilizados.

Após a pesquisa em todas as bases de dados, os artigos encontrados foram migrados para o *software Rayyan* ^®^ versão *Web* gratuita, possibilitando a análise automática e manual de duplicatas. Com o auxílio dessa ferramenta, dois pesquisadores puderam, de forma cega e independente, realizar a avaliação em duas etapas: (1) leitura do título e resumo e (2) leitura do texto completo. A cada etapa, as divergências foram resolvidas por meio de consenso entre os dois pesquisadores conforme recomendado em literatura, não sendo necessário utilizar um terceiro^(14)^.

Os dados dos artigos incluídos foram extraídos usando uma planilha própria do *software Microsoft Excel* ^®^ *,* contendo informações específicas sobre título, autores, ano, país de origem, revista de publicação, idioma, objetivo do estudo, abordagem metodológica, população estudada (usuários com hipertensão e diabetes), número de participantes, contexto (APS e AEA), definição de coordenação do cuidado e principais resultados.

### Tratamento e análise dos dados

Após os dados serem extraídos, foi utilizada a Análise de Conteúdo, do tipo temático-categorial, que, de acordo com Minayo^(15)^, é quando se descobrem os núcleos de sentido daquela comunicação cuja frequência ou presença signifique algo para o objetivo do estudo. Ainda foram utilizadas as três etapas recomendadas: pré-análise, com a organização e preparação do material; exploração do material, com a codificação dos dados por meio das unidades de registro; e o tratamento dos resultados, interpretação e inferências^(16)^.

Para a segunda etapa, com exploração do material, utilizou-se o *software* ATLAS.ti *Web*, versão paga, que facilita a criação de categorias. A utilização do *software* ocorreu em dois momentos: primeiro, o conteúdo retirado das referências foi separado por tópicos-chave que representassem a essência do que foi descrito e que respondiam à pergunta da pesquisa. Após este momento, os tópicos-chave foram agregados, formando um grupo de categorias que tivessem similaridade, representando os conceitos e características da coordenação do cuidado.

Com a categorização realizada por meio do *software* informado, foi possível analisar em cada artigo quais categorias estiveram mais prevalentes, realizando um cruzamento das informações após o tratamento dos resultados.

### Aspectos éticos

Considerando que este estudo não realizou avaliação com seres humanos, mas sim com informações secundárias de acesso e domínio público, exclusivamente por meio de artigos científicos, não foi necessária a submissão ao Comitê de Ética em Pesquisa, de acordo com a Resolução do Conselho Nacional de Saúde de nº 466, de 12 de dezembro de 2012, e suas complementares.

## Resultados

A pesquisa resultou inicialmente em 1.506 publicações, das quais 943 foram identificadas como duplicatas, restando 563 publicações para leitura de títulos e resumos. Desses, 149 foram elegíveis para leitura de texto completo, que, com a aplicação dos critérios de exclusão, resultaram em 40 artigos incluídos nesta revisão, conforme processo de seleção detalhado na Figura 1.

Na Figura 2, é apresentada a descrição dos artigos que foram incluídos como amostra final desta revisão de escopo.

Em relação ao ano de publicação dos artigos selecionados, eles datam desde o ano 2000, com anos sequenciais de publicação encontradas a partir de 2009 até 2022. Em relação aos países de origem dos artigos, eles foram realizados nos Estados Unidos (n=25)^(17,19-24,27-29,31-33,35,38,40-43,45-49,53)^, seguido pelo Brasil (n=3)^(36-37,56)^, Austrália (n=3)^(30,34,51)^ e pelos demais oito países, com um ou dois artigos, quais sejam: África do Sul, País Basco, Canadá, Coreia do Sul, Índia, Itália, Noruega e Taiwan.

**Figura 1:** - Fluxograma do processo de seleção dos estudos para a revisão de escopo adaptado do *Preferred Reporting Items for Systematic Review and Meta-Analyses* (PRISMA)^(13)^

**Figura 2:** - Quadro de descrição dos artigos selecionados por autor/referência, ano, título, abordagem metodológica e categoria populacional

**Autor / Referência**	**Ano**	**Título**	**Abordagem metodológica / Desenho do estudo**	**População estudada**
Schillinger, et al.^(17)^	2000	*Effects of primary care coordination on public hospital patients*	Quantitativo / Randomizado	Usuário com hipertensão e diabetes
Mills; Harvey^(18)^	2003	*Beyond community* - *based diabetes management and the COAG coordinated care trial*	Quantitativo / Caso controle	Usuário com diabetes
O’Malley; Cunningh^(19)^	2009	*Patient experiences with coordination of care: the benefit of continuity and primary care physician as referral source*	Quantitativo / Estudo Transversal	Usuário com hipertensão e diabetes
MacPhailet, et al.^(20)^	2009	*Coordination of diabetes care in four delivery models using an electronic health record*	Qualitativo / Estudo de caso múltiplo	Usuário com diabetes
Liu, et al.^(21)^	2010	*Care Fragmentation and Emergency Department Use Among Complex Patients With Diabetes*	Quantitativo / Observacional	Usuário com diabetes
Hummel; Gandara^(22)^	2011	*Health information exchange and care coordination of diabetic patients between medicine and dentistry*	Qualitativo / Estudo de caso	Usuário com diabetes
Maeng, et al.^(23)^	2012	*Care coordination for the chronically ill: understanding the patient’s perspective*	Quantitativo / Transversal	Usuário com hipertensão e diabetes
Pollack, et al.^(24)^	2013	*Patient sharing among physicians and costs of care: a network analytic approach to care coordination using claims data*	Quantitativo / Coorte	Usuário com diabetes
Baldo, et al.^(25)^	2014	*Diabetes outcomes within integrated healthcare management programs*	Quantitativo / Observacional	Usuário com diabetes
Katz, et al.^(26)^	2014	*Do primary care physicians coordinate ambulatory care for chronic disease patients in Canada?*	Quantitativo / Coorte	Usuário com hipertensão e diabetes
Liss, et al.^(27)^	2014	*Specialty use among patients with treated hypertension in a patient-centered medical home*	Quantitativo / Estudo de série temporal	Usuário com hipertensão
Segal; Dugoff^(28)^	2014	*Building blocks for measuring care coordination with claims data*	Qualitativo / Observacional	Usuário com diabetes
Weeks, et al.^(29)^	2014	*Measuring Primary Care Organizational Capacity for Diabetes Care Coordination: The Diabetes Care Coordination Readiness Assessment*	Quantitativo	Usuário com diabetes
Dawda, et al.^(30)^	2015	*Does it matter who organises your health care?*	Quantitativo	Usuário com diabetes
Haley, et al.^(31)^	2015	*Improving Care Coordination Between Nephrology and Primary Care: A Quality Improvement Initiative Using the Renal Physicians Association Toolkit*	Quantitativo / Estudo longitudinal	Usuário com hipertensão e diabetes
Wang, et al.^(32)^	2015	*Association of patient-reported care coordination with patient satisfaction*	Quantitativo / Estudo Transversal	Usuário com diabetes
Zlateva, et al.^(33)^	2015	*Development and validation of the Medical Home Care Coordination Survey for assessing care coordination in the primary care setting from the patient and provider perspectives*	Quantitativo	Usuário com hipertensão e diabetes
Lo, et al.^(34)^	2016	*Primary and tertiary health professionals’ views on the health-care of patients with co-morbid diabetes and chronic kidney disease - a qualitative study*	Qualitativo	Usuário com diabetes
Malkani, et al.^(35)^	2016	*Redesigning Diabetes Care: Defining the Role of Endocrinologists Among Alternative Providers*	Qualitativo	Usuário com diabetes
Venancio, et al.^(36)^	2016	Atenção integral à hipertensão arterial e diabetes mellitus: implementação da Linha de Cuidado em uma Região de Saúde do estado de São Paulo, Brasil	Misto / Estudo de caso	Usuário com hipertensão e diabetes
Aleluia, et al.^(37)^	2017	Coordenação do cuidado na atenção primária à saúde: estudo avaliativo em município sede de macrorregião do nordeste brasileiro	Qualitativo / Estudo de caso	Usuário com hipertensão e diabetes
Fitzgerald, et al.^(38)^	2017	*Program Implementation Approaches to Build and Sustain Health Care Coordination for Type 2 Diabetes*	Qualitativo	Usuário com diabetes
Provost, et al.^(39)^	2017	*Implementation of an integrated primary care cardiometabolic risk prevention and management network in Montréal: Does greater coordination of care with primary care physicians have an impact on health outcomes?*	Misto	Usuário com hipertensão e diabetes
Talley, et al.^(40)^	2018	*Improving Population Health among Uninsured Patients with Diabetes*	Quantitativo / Estudo observacional	Usuário com diabetes
Van-Eeghen, et al.^(41)^	2018	*Chronic care coordination by integrating care through a team-based, population-driven approach: a case study*	Qualitativo / Estudo de Caso	Usuário com diabetes
Vimalananda, et al.^(42)^	2018	*Patient, Primary Care Provider, and Specialist Perspectives on Specialty Care Coordination in an Integrated Health Care System*	Qualitativo	Usuário com diabetes
Benzer, et al.^(43)^	2019	*Survey of Patient-Centered Coordination of Care for Diabetes with Cardiovascular and Mental Health Comorbidities in the Department of Veterans Affairs*	Quantitativo / Estudo Observacional	Usuário com hipertensão e diabetes
Lee; Bae^(44)^	2019	*Implementation of a care coordination system for chronic diseases*	Qualitativo	Usuário com hipertensão e diabetes
McLendo, et al.^(45)^	2019	*Enhancing diabetes care through care coordination, telemedicine, and education: Evaluation of a rural pilot program*	Quantitativo / Coorte	Usuário com diabetes
Mohr et al.^(46)^	2019	*Organizational Coordination and Patient Experiences of Specialty Care Integration*	Misto / Estudo transversal	Usuário com hipertensão e diabetes
Williams, et al.^(47)^	2019	*Sustainable care coordination: a qualitative study of primary care provider, administrator, and insurer perspectives*	Qualitativo	Usuário com diabetes
Benzer, et al.^(48)^	2020	*A Mixed Methods Study of the Association of Non-Veterans Affairs Care With Veterans’ and Clinicians’ Experiences of Care Coordination*	Misto / Estudo Observacional	Usuário com hipertensão e diabetes
Harrison, et al.^(49)^	2020	*Economic outcomes of insurer-led care management for high-cost Medicaid patients*	Quantitativo / Coorte	Usuário com hipertensão e diabetes
Mateo-Abad, et al.^(50)^	2020	*Impact of the CareWell integrated care model for older patients with multimorbidity: a quasi-experimental controlled study in the Basque Country*	Misto / Estudo quase-experimental	Usuário com diabetes
Blignault, et al.^(51)^	2021	*“You Can’t Work with My People If You Don’t Know How to”: Enhancing Transfer of Care from Hospital to Primary Care for Aboriginal Australians with Chronic Disease*	Qualitativo	Usuário com diabetes
Chen; Cheng^(52)^	2021	*Care Continuity and Care Coordination: A Preliminary Examination of Their Effects on Hospitalization*	Quantitativo / Coorte	Usuário com diabetes
Cook, et al.^(53)^	2021	*Registry-Managed Care Coordination and Education for Patients With Co-occurring Diabetes and Serious Mental Illness*	Quantitativo / Coorte	Usuário com diabetes
Helmersen, et al.^(54)^	2021	*Women’s experience with receiving advice on diet and Self-Monitoring of blood glucose for gestational diabetes mellitus: a qualitative study*	Qualitativo	Usuário com diabetes
Jindal, et al.^(55)^	2022	*Improving care for hypertension and diabetes in India by addition of clinical decision support system and task shifting in the national NCD program: I-TREC model of care*	Qualitativo / Estudo de caso	Usuário com hipertensão e diabetes
Rêgo, et al.^(56)^	2022	Coordenação do cuidado na perspectiva das pessoas com hipertensão na atenção primária à saúde	Quantitativo / Estudo transversal	Usuário com hipertensão

Os artigos foram publicados em 26 revistas científicas diferentes, com concentração maior de artigos na revista *Journal of General Internal Medicine* (n=7)^(17,19,24,27,29,43,36)^, seguido da revista BMC *Health Services Research* (n=5)^(23,33,47,50,55)^. Em relação ao idioma dos artigos, foram publicados em inglês (n=37)^(17-35,38-55)^ e português (n=3)^(36-37,56)^. No que se refere à abordagem metodológica utilizada nos artigos, grande parte deles definiram-se como quantitativos (n=21)^(17-19,21,23-27,29-33,40,43,45,49,52-53,56)^, seguido de qualitativos (n=14)^(20,22,28,34-35,37-38,41-42,44,47,51,54-55)^ e mistos (n=5)^(36,39,46,48,50)^.

Algumas definições de coordenação do cuidado foram identificadas nos artigos selecionados, diante disso, para descrever de forma sistematizada, foi realizada uma análise do conteúdo abordado, identificando no texto as características mais citadas por meio da categorização.

Foram elencados 108 tópicos-chave, divididos em 16 categorias. No entanto, para facilitar a compreensão, e pela proximidade das temáticas, foram agrupados em oito categorias conforme mostra o gráfico de funil, na Figura 3, as quais serão apresentadas a seguir, em uma síntese de cada tópico, com as características apontadas pelos artigos, para que o conceito de coordenação do cuidado possa ser entendido em sua ampla dimensão.

**Figura 3:** - Gráfico de funil com as definições da coordenação do cuidado por categorias

### Informação e comunicação

Os sistemas de registros tecnológicos são necessários para viabilizar o reconhecimento e transferência de informações entre os diversos fornecedores de serviços, assegurando uma comunicação eficaz, com vistas a facilitar o cuidado coordenado, inclusive entre a equipe de cuidados primários e os demais especialistas^(22-24,26,28,32-33,38,41,46,48,52-53)^.

### Integração do cuidado

A coordenação do cuidado é uma estratégia de prestação de cuidados de saúde responsável por integrar os serviços e as ações de saúde dos usuários de forma complementar dentro dos diferentes níveis de atenção. Para isso, é imperativa sua estruturação, uma vez que representa um componente essencial na regionalização dos serviços nos sistemas de saúde^(22,26,28,30,37,47,52,56)^.

### Melhoria e qualidade

Coordenar o cuidado significa desempenhar um papel fundamental na promoção de melhorias e na entrega apropriada de cuidados aos usuários, promovendo um aumento na qualidade da prestação de serviços de maneira organizada, eficaz e eficiente. Esse processo propicia a melhoria dos desfechos na área da saúde, a redução dos custos associados, devido à otimização da utilização de serviços, e em melhorias significativas nos resultados clínicos^(20,23,26,32,37-38,43,46-47,53,56)^.

### Gestão do cuidado

Na coordenação do cuidado, o planejamento e organização da assistência pode ser efetivado por meio da padronização de processos e de procedimentos entre as partes. Neste sentido, realizar a gestão das necessidades dos pacientes e monitorar os planos de cuidado, gerando responsabilização, são características essenciais para que sejam gerenciados os serviços que os usuários realmente necessitam, reorientando as demandas quando necessário e antecipando as necessidades futuras^(19,22,29,33,37-38,41,43,46,52-53,56)^.

### Compartilhamento do cuidado

O mecanismo de referência e contrarreferência, em conformidade com as melhores práticas na área da saúde, viabiliza a interdependência e a adequada condução do cuidado oferecido entre as distintas instâncias de serviços de saúde, mediante uma transição de cuidado oportuna e segura. Esse processo se efetiva quando múltiplos intervenientes, incluindo o próprio usuário, os profissionais de cuidados primários e os demais prestadores de serviços colaboram e compartilham a responsabilidade pelos cuidados^(20,22-23,29-30,33,37-38,41-42,44,48,52)^.

### Atributo fundamental

Amplamente reconhecido como um atributo fundamental, a coordenação do cuidado tem notável importância, caracterizando-se por ser essencial para a APS e por desempenhar um papel crítico na promoção do acesso aos serviços de saúde. Assegura a continuidade do cuidado, minimizando barreiras de acesso e estabelecendo a conexão entre os recursos comunitários, contribuindo para a provisão de cuidados centrados no paciente^(19-20,26,30,37-38,52)^.

### Profissionais da saúde

Há o envolvimento de uma extensa variedade de profissionais de saúde e recursos para se operacionalizar a coordenação do cuidado. Nesse sentido, para que a execução das atividades essenciais e cuidados necessários aos usuários sejam viáveis, é imperativo o estabelecimento de uma sólida conexão entre as partes e a promoção de um trabalho eficiente entre as equipes prestadoras dos cuidados em saúde^(23,26,37-38,46,53)^.

### Usuários dos serviços de saúde

Apesar de a coordenação do cuidado ter sido empregada para atender a todos usuários dos serviços de saúde, no contexto das condições crônicas, surge como uma estratégia de resposta que deve ser estimulada em toda a sua abrangência, especialmente em virtude dos usuários que recebem cuidados de diversos prestadores em múltiplos ambientes, já que isto resulta em um incremento nos custos associados a esses cuidados e a riscos clínicos^(26,30,32,41,47,56)^.

No que se refere aos resultados apresentados pelos artigos, foram consideradas as categorias supracitadas, exceto a categoria atributo fundamental, com o objetivo de relacionar as principais características dos resultados às características conceituais apontadas sobre a coordenação do cuidado. Destarte, por meio do *software* ATLAS.ti *Web*, realizou-se o cruzamento das informações identificadas nos principais resultados de cada artigo para identificar neles as categorias mais presentes, por meio do Diagrama de Sankey, conforme Figura 4.

**Figura 4:** - Diagrama de Sankey das categorias dos resultados dos estudos por artigo selecionado

Os resultados dos artigos se concentraram principalmente em quatro categorias, tendo destaque a categoria de informação e comunicação, seguida da categoria de gestão do cuidado e da categoria de compartilhamento do cuidado em paralelo com melhoria e qualidade.

No que se refere à categoria *informação e comunicação*, a temática tem se mostrado um dos desafios centrais da coordenação de cuidados, já que as informações prestadas não apresentam detalhes suficientes para a tomada de decisão. No entanto, com a integração do prontuário eletrônico, a gestão da informação foi aprimorada e, ainda, melhorias no prontuário eletrônico, como a sinalização de *feedback*, geraram melhora na coordenação do cuidado^(22,37-38,46-48,53)^.

Nesse sentido, foi relatado que o encaminhamento por meio de um sistema eletrônico simplifica o registro das informações e se mostra uma ferramenta usual para o acompanhamento do usuário, apesar de o próprio usuário, em alguns momentos, não identificar que o profissional está realizando as anotações^(20,45,55-56)^.

Em contrapartida, a coordenação do cuidado requer mais do que o estabelecimento de recursos eletrônicos, além disso, as fichas de referência e contrarreferência não têm apresentado muito êxito, tendo em vista que foram pouco utilizadas na contrarreferência^(20,22,29,32,37)^. Vale ressaltar, que um sistema eletrônico, dependendo da estrutura necessária, pode ter custos elevados para as organizações, sendo de difícil implementação, principalmente devido aos recursos limitados^(35,41)^.

Em relação à *gestão do cuidado*, os usuários que recebem atendimento do médico de família e outros médicos especialistas apresentam resultados de saúde melhores do que aqueles prestados somente pelo médico de família, reduzindo a utilização hospitalar e os custos para o sistema de saúde^(17,25,39,45)^. Ainda, do ponto de vista dos usuários, a coordenação do cuidado é muito melhor quando realizada por um profissional de cuidados primários específico, sendo também importante a decisão desse profissional em relação a qual especialista consultar^(19,28)^.

No que se refere ao *compartilhamento do cuidado,* os mecanismos formais de referência e contrarreferência têm se mostrado insuficientes, principalmente devido à ausência de contrarreferência dos níveis secundários e terciários, e inclusive para o tratamento de usuários com diabetes e hipertensão. No entanto, o aumento do encaminhamento dos usuários de alta morbidade para especialistas destaca a necessidade de uma coordenação mais eficiente^(27,36-37)^.

Foi evidenciado, inclusive, que quando um médico não é o responsável pelo cuidado do usuário, é mais provável que ele realize um encaminhamento para a atenção especializada por falta de informações e por não conhecer o histórico do usuário, o que é diferente de quando realizado pelos profissionais de cuidados primários^(17,26)^.

No que se refere à *melhoria e qualidade*, foi apontado que a coordenação do cuidado deve ser uma prioridade, tendo em vista que ela garante a qualidade do atendimento, gerando resultados de saúde melhores aos usuários^(18,39,45,48)^. Além disso, para garantir um cuidado mais adequado e planejado, a transferência do cuidado deve levar em consideração as questões culturais dos usuários^(51)^.

Nesse sentido, para se realizar um cuidado coordenado na perspectiva da melhoria do financiamento dos sistemas de saúde, estudos evidenciaram que pacientes com diabetes e insuficiência cardíaca congestiva, assim como aqueles com elevados riscos, que receberam cuidados compartilhados por meio da coordenação do cuidado, apresentaram menores custos totais, menor taxa de internação e hospitalização^(24)^.

Outro estudo, apesar de considerar que a coordenação poderia ter reduzido os custos totais e/ou farmacêuticos, não encontrou evidências significativas^(49)^. Ainda em relação a custos, foi evidenciado que a coordenação do cuidado, em sistemas privados de saúde, pode ser cobrada aos usuários, levando à falta de participação dos usuários em função dos altos custos^(47)^.

No que concerne à categoria *profissionais da saúde*, os usuários e os seus cuidadores percebem que eles são bons como coordenadores do cuidado, tal concepção é ratificada, ainda, pela percepção dos médicos especialistas e de cuidados primários^(34,50)^. Não obstante, foi apontado que usuários com uma ou mais visitas aos médicos de família nos últimos três meses têm níveis de coordenação mais elevados^(30)^.

Foi identificado, em contraponto, que há indicações de que ter um enfermeiro como gestor de cuidados pode melhorar a coordenação do cuidado, inclusive na percepção dos usuários, que também incluíram papel importante dos farmacêuticos e dos Agentes Comunitários de Saúde nesse processo, principalmente por meio da busca ativa dos usuários nas visitas domiciliares^(30,37,42)^.

Em relação aos *usuários dos serviços de saúde,* aqueles com condições crônicas e que se consultam com o mesmo médico apresentam uma melhora na adesão do tratamento, com resultados significativos, devido ao estabelecimento de vínculo e à continuidade do cuidado^(30,36)^.

Nesse sentido, foi evidenciado que usuários com diabetes acompanhados apenas no nível primário possuem taxa de mortalidade mais elevadas do que os compartilhados com o serviço especializado, com aumento expressivo nos usuários em uso de insulina^(25)^. No entanto, apesar de apresentarem menor probabilidade de sofrer hospitalização, os pacientes com baixa morbidade são encaminhados em menor quantidade devido às suas necessidades serem sanadas no âmbito da atenção primária^(27,52)^.

Ainda no que se refere às condições crônicas, conforme apontado por estudo, quando o usuário apresenta maior gravidade, como nos casos de diabetes tipo 2, a fragmentação encontrava-se mais presente, sugerindo que a gravidade aumenta os desafios da coordenação^(47)^. Apesar disso, foi relatado que pessoas com condições mais complexas têm coordenação do cuidado mais consistente^(30)^.

Estudos sobre os usuários dos serviços de saúde mostram que aqueles acima de 60 anos têm níveis de coordenação mais elevados do que os com idade inferior, bem como usuários mais ativos são menos propensos a relatarem problemas de coordenação por serem melhores autogestores dos seus cuidados, e aqueles usuários que são mais complexos se beneficiam de maiores esforços quanto à coordenação do cuidado^(23,30)^.

Em relação aos usuários com hipertensão, aqueles com a pressão arterial controlada tendem a avaliar positivamente a coordenação do cuidado, enquanto os descompensados a avaliam de forma insatisfatória^(56)^. Assim, percebe-se que quanto maior a gravidade da condição do paciente, maior será a probabilidade de ele necessitar de outro serviço e especialidades^(44)^.

Relativo à *integração do cuidado,* faz-se necessária maior proximidade entre as equipes nos modelos organizacionais da atenção primária, principalmente nos casos complexos de diabetes, no qual há fragmentação do cuidado^(21,39)^. Nessas situações, a continuidade de atendimento pelos mesmos profissionais é benéfica e pode favorecer, inclusive, no esclarecimento dos papéis dos profissionais no cuidado, o que melhora a coordenação do cuidado^(19)^.

## Discussão

É evidente que a coordenação do cuidado para as condições crônicas, em específico para HAS e DM, caracteriza-se por ser um atributo fundamental, gerando integração e compartilhamento dos cuidados dos usuários, por meio de uma gestão do cuidado que garante melhoria e qualidade na assistência e no sistema de saúde, necessitando, para sua operacionalização, de excelentes meios de informação, comunicação e profissionais capacitados.

Nesse sentido, diversos têm sido os esforços para investigar e compreender os fatores, perspectivas e desafios que os modelos de sistemas de saúde universais vêm realizando para se manter sustentáveis. No entanto, sabe-se que os hospitais possuem papel protagonista e, ao mesmo tempo, são produtos da fragmentação do cuidado. Frente a isso, espera-se que sejam produzidas conexões entre os serviços de saúde, inclusive junto a APS^(57)^.

É fato que todos os sistemas têm desafios similares frente ao atendimento efetivo, eficiente e equitativo, mesmo que apresentem diferentes características e formas de serem operados, como no caso do Brasil e dos Estados Unidos da América (EUA). Ainda assim, todos têm trabalhado para responder às dificuldades de uma forma que não gere problemas econômicos, mas que auxilie na redução dos gastos com os serviços de saúde, mesmo com as ideologias diferentes de cada sistema^(58-59)^.

Assim, a coordenação do cuidado entre a APS e a AEA vem sendo discutida inclusive nos EUA que, mesmo não tendo um sistema de saúde universal, tenta identificar a experiência e percepção dos usuários frente à coordenação, avaliar os resultados médicos dos usuários com a coordenação do cuidado e avaliar se há diminuição dos custos hospitalares devido à coordenação^(23-24,48)^.

Acredita-se que a coordenação do cuidado seja responsável por promover melhorias na qualidade da assistência aos usuários, reduzir barreiras de acesso e integrar as ações e serviços nos sistemas de saúde, além disso, são amplas as definições dadas para este atributo, conforme apontado por este estudo. Assim, é fato que quanto maior a quantidade de pessoas e serviços envolvidos no cuidado e quanto mais complexa for a intervenção, maior é o nível de coordenação necessário para se alcançar os resultados desejados^(3)^.

Diante do exposto, foi evidenciado que, para garantir uma coordenação do cuidado em sua plenitude, faz-se necessária uma efetiva informação e comunicação, sendo assim realizada por meio de sistemas de registros tecnológicos com a transferência e intercambialidade das informações dos usuários.

Nesse sentido, entende-se o Registro Eletrônico de Saúde (RES) como uma tecnologia que orienta as necessidades dos usuários, unindo as informações de um indivíduo ou de um grupo e compartilhando essas informações entre as instituições. Além disso, o RES pode facilitar os serviços de saúde no monitoramento da situação de saúde e gestão financeira por meio de relatórios. No entanto, a implementação desses sistemas possui desafios, como o desenvolvimento de *software*, custo-efetividade, armazenamento dos dados e o desempenho dos programas^(60)^.

Um estudo realizado descobriu que a implementação do Prontuário Eletrônico do Paciente (PEP), utilizado pela APS no Brasil, favoreceu a organização da rede de serviços com integração horizontal entre as equipes, além disso, influenciou na corresponsabilização do cuidado e na produção da autonomia dos profissionais de saúde^(60)^. O autor relata, também, que há relevância no uso do PEP para a APS ao favorecer a coordenação do cuidado, a integralidade da assistência e a longitudinalidade, o que corrobora o evidenciado nos estudos encontrados nesta revisão.

No entanto, apesar dos pontos positivos no uso do RES, ainda há resistência por parte dos profissionais, que pode estar relacionada à falta de capacitação para o seu uso. Isto foi evidenciado, pois, mesmo nos locais onde o prontuário eletrônico já está implantado, ele se encontra subutilizado^(60)^.

Em outro estudo^(61)^, foi relatado que alguns profissionais da saúde não gostaram da ideia de se implantar prontuário eletrônico, já que muitos não sabiam utilizá-lo e não possuíam conhecimento sobre equipamentos tecnológicos. Apesar disso, com o uso, eles começaram a ter um comportamento positivo sobre a ferramenta, principalmente após as capacitações realizadas.

Em consonância com o que foi relatado nos estudos desta revisão, compreende-se que o RES são necessários para organização e reestruturação de um sistema de saúde, já que sua ausência fragiliza a referência e a contrarreferência, inibindo a troca de informações entre a APS e os outros serviços de saúde. Diante disso, é necessário integrar os registros de prontuário, de forma a acessar as condutas, exames e diagnósticos realizados pelos profissionais para que, assim, seja feita uma gestão dos processos e fluxos^(61-62)^.

Em relação à gestão do cuidado, os estudos encontrados nesta revisão abordaram e identificaram que, para se realizar a coordenação do cuidado, é necessário planejamento e organização da assistência com a padronização de processos e procedimentos.

Diante disso, para se organizar os sistemas de saúde, a gestão do cuidado se insere em um novo paradigma, já que os usuários têm apresentado necessidades complexas de saúde, requerendo maior capacidade de gestão. No entanto, isso somente é possível por meio das relações entre os integrantes, com foco principal no usuário, tendo a APS um caráter complexo dentre os serviços de saúde^(63)^.

Dessa forma, para que se fortaleçam as conexões, os profissionais precisam se preocupar com a literacia para saúde, também conhecida como letramento em saúde, tendo em vista que os usuários necessitam processar e compreender as informações básicas sobre saúde, incluindo, o conhecimento sobre o funcionamento do sistema de saúde e da coordenação do cuidado, inclusive para os usuários com DCNT, por serem doenças com elevada complexidade^(64)^.

Nesse sentido, alguns autores^(65)^ relataram que, na atenção às condições crônicas, faz-se necessária a reorganização dos processos de trabalho, com melhoria no planejamento e integração entre os membros da equipe, para que seja produzido um novo modo de gestão do cuidado em saúde.

Diante disso, sabe-se que, para se realizar a gestão do cuidado dos usuários, principalmente no que diz respeito àqueles com condições crônicas, os sistemas de saúde precisam de uma atenção especializada (ambulatorial e hospitalar) fortalecida, o que tem sido um dos maiores problemas do SUS, com ofertas insuficientes para se realizar o que é necessário aos usuários^(66)^.

Além disso, os artigos avaliados nesta revisão não abordam o papel da AEA na coordenação do cuidado, bem como não é abordada a contrarreferência dos usuários com hipertensão e diabetes para a APS; dessa forma, mostra-se como uma lacuna, que talvez ocorra, pois, conforme demonstrado o apoio da AEA é insuficiente, não tendo sido ainda transpassado esta barreira o que impossibilita outros avanços.

Salienta-se que a recente normativa publicada no Brasil por meio da Portaria nº 1.604, de 18 de outubro de 2023, do Ministério da Saúde, que institui a Política Nacional de Atenção Especializada em Saúde, é um marco norteador para o SUS. Diante disso, espera-se que, com o fortalecimento da AEA, ocorra uma melhora na coordenação do cuidado, garantindo, assim, melhor apoio à APS.

Em relação ao compartilhamento do cuidado, os estudos desta revisão trouxeram que, para que a coordenação do cuidado seja efetivada, é necessário que o compartilhamento seja feito com responsabilidade, gerando interdependência e adequada condução do cuidado. Destacam, ainda, que o compartilhamento do cuidado deve ocorrer de forma oportuna e segura.

Em vista disso, um estudo que aborda o encaminhamento de usuários ao cardiologista e ao endocrinologista concluiu que, apesar dos médicos da APS utilizarem os protocolos de encaminhamento, ainda seriam necessárias adequações, pois estes, por si só, não são capazes de solucionar a problemática devido à falta de recursos diagnósticos^(67)^.

Ressalta-se que, com melhorias na implementação de sistema de prontuário único, *telecharts* e reuniões capacitadoras, o compartilhamento do cuidado poderia ser adequado, o que evitaria os encaminhamentos desnecessários, reduzindo filas e custos, e aumentando o acesso dos usuários a AEA^(67)^.

Destaca-se que a coordenação do cuidado representa uma estratégia para integração dos níveis de atenção, sendo impreterível para reorientar os serviços de saúde e as necessidades dos usuários. Diante disso, envolve a criação e a manutenção de uma estrutura comum, que tem o propósito de coordenar sua interdependência para permitir um trabalho coletivo^(68)^.

Ainda, um estudo que considerou o impacto das DCNTs na morbimortalidade e nos custos para os sistemas de saúde reconheceu a importância da enfermagem^(68)^, já que são conferidos ao profissional enfermeiro a capacidade resolutiva, o protagonismo e a autonomia, bem como a capacidade de coordenação, permitindo o direcionamento mais assertivo dos usuários pelos serviços de saúde, conforme foi apontado por estudos desta revisão.

Em suma, nota-se que, com os resultados apresentados, o conceito e as características da coordenação do cuidado se mostram mais claros, o que pode auxiliar na ampliação da avaliação desse atributo por meio de indicadores e na melhora da tomada de decisão, inclusive para a assistência de usuários com DCNT, além de que é fato que este atributo precisa ser entendido, discutido e aplicado dentro dos sistemas de saúde em toda sua magnitude.

Destarte, esta revisão evidencia a importância da coordenação do cuidado para os sistemas de saúde, bem como fica claro que a coordenação possui uma definição ampla e complexa. No entanto, alguns pontos se mostraram essenciais para o avanço no entendimento deste tema, sendo necessário o aprimoramento da informação e da comunicação, da gestão e do compartilhamento do cuidado.

Esta revisão de escopo apresenta como limitação o fato do termo “coordenação do cuidado” não estar definido como um descritor DeSC/MeSH, sendo assim necessária para a busca deste tema uma combinação de outros descritores.

No que se refere às lacunas encontradas, foi observado que há pouca informação e/ou informações divergentes na literatura em relação aos custos dos sistemas de saúde que apresentam ou não estruturação na coordenação do cuidado. Além disso, é notória a necessidade de mais estudos que avaliem a coordenação do cuidado sob a perspectiva da AEA, ficando evidentes somente o papel e os desafios da APS para que os usuários cheguem até a AEA, não sendo abordado o cuidado neste nível de atenção e os desdobramentos deste serviço até o retorno dos usuários à APS.

## Conclusão

Esta revisão mostrou que os estudos tiveram como principais resultados a informação e comunicação, a gestão do cuidado e o compartilhamento do cuidado como categorias da coordenação do cuidado, diante disso, entende-se que são características marcantes que devem ser investigadas durante a avaliação desse atributo.

Com os avanços tecnológicos e com a rapidez das informações, observa-se que ferramentas tecnológicas são um primeiro passo na garantia da coordenação do cuidado, que se mostraram uma característica muito significativa entre os estudos desta revisão, com ênfase nos estudos sobre o compartilhamento de informações entre os serviços de saúde por meio de prontuários eletrônicos. No entanto, apesar dessa tecnologia se mostrar muito vantajosa para o sistema de saúde, com bons resultados, ela não é o único meio de se garantir a coordenação do cuidado.

No que se refere às DCNTs, por se tratarem de doenças complexas que necessitam de diversos profissionais para a realização do cuidado adequado, é fundamental que a coordenação do cuidado seja efetiva, visto ter se mostrado positiva e vantajosa, quando executada. Nesse sentido, fica evidente que a APS é o principal e mais capacitado serviço dentro do sistema de saúde para realizar a coordenação do cuidado.

No entanto, em relação a AEA, são necessários mais estudos que abordem o serviço de saúde em relação à coordenação do cuidado, já que tem demonstrado fragilidades dentro do sistema de saúde. Ainda, ressalta-se que a APS, por si só, não é capaz de resolver todas as necessidades de saúde dos usuários, mesmo sendo resolutiva em grande parte das demandas.

Esta revisão de escopo não possui conflito de interesses e não recebeu financiamento externo para sua realização.
